# Signal-Driven Model Order Selection for MUSIC-Based HRV Spectral Characterization

**DOI:** 10.3390/bioengineering13091033

**Published:** 2026-09-05

**Authors:** Perla Lizeth Garza-Barrón, Alejandro Barrientos-García, Carlos Mauricio Lastre-Domínguez, Claudia Angélica Rivera-Romero, Juvenal Villanueva-Maldonado, Jorge Ulises Muñoz-Minjares

**Affiliations:** 1 Posgrado en Ingeniería para la Innovación Tecnológica, Universidad Autónoma de Zacatecas, Carr. Zacatecas–Guadalajara Km. 6, Ejido “La Escondida”, Zacatecas 98160, Mexico; lizeth2408@uaz.edu.mx; 2 Óptica no lineal y Procesamiento de Señales, Ingeniería Robótica, Universidad Politécnica de Guanajuato (UPG), Avenida Universidad Sur No. 1001, Cortazar 38496, Mexico; abarrientos@upgto.edu.mx; 3 División de Estudios de Posgrado e Investigación, Instituto Tecnológico de Oaxaca, Tecnológico Nacional de México, Avenida Ing. Víctor Bravo Ahuja No. 125 Esquina Calzada Tecnológico, Oaxaca de Juárez 68030, Mexico; carlos.lastre@itoaxaca.edu.mx; 4 Unidad Académica de Ingeniería Eléctrica, Universidad Autónoma de Zacatecas, Carretera Zacatecas–Guadalajara Km. 6, Zacatecas 98160, Mexico; c.a.riveraromero@uaz.edu.mx (C.A.R.-R.); juvenal.villanueva@uaz.edu.mx (J.V.-M.)

**Keywords:** heart rate variability, dominant frequency estimation, MUSIC algorithm, autonomic modulation, spectral characterization, emotional stimulation

## Abstract

Heart rate variability (HRV) is a useful non-invasive tool for studying autonomic nervous system modulation under emotional stimulation; however, accurate estimation of dominant frequencies in HRV signals remains challenging due to their non-stationary nature and the sensitivity of some spectral methods to configuration parameters. This work presents a methodology for the spectral characterization of HRV signals derived from ECG recordings from the DREAMER database, with emphasis on optimizing the model order of the MUSIC algorithm to improve dominant frequency localization within the physiological low-frequency (LF) and high-frequency (HF) bands. The proposed methodology included ECG signal preprocessing, R-peak detection, RR interval extraction, HRV interpolation, and spectral analysis using MUSIC, while evaluating different model orders through a signal-driven composite criterion based on AIC, MDL, ESTER, eigengap, and model complexity. The criteria were normalized using min–max normalization and combined using equal predefined weights. The results showed that the signal-driven selection of the parameter *p* produced recording-dependent model order configurations and different dominant frequency estimates across the analyzed stimuli. The resulting LF/HF agreement was evaluated independently after model order selection and showed non-uniform correspondence across stimuli and spectral estimators. Overall, these findings indicate that model order selection can substantially influence the spectral characterization obtained with MUSIC and provide a signal-driven framework for examining this dependence in HRV recordings.

## 1. Introduction

Emotions are accompanied by physiological changes mediated by the autonomic nervous system (ANS), which has led to growing interest in their study across fields such as biomedical engineering, psychophysiology, and affective computing [1,2]. Recent studies have shown that the analysis of physiological signals supported by advanced processing techniques can contribute to a more robust characterization of affective responses [3]. Among the different physiological signals used to study these responses, electrocardiographic (ECG) recordings constitute an objective and non-invasive tool for evaluating cardiac dynamics and their relationship with autonomic modulation [4,5]. From ECG recordings, it is possible to derive heart rate variability (HRV), which reflects the interaction between the sympathetic and parasympathetic branches of the ANS and has been widely used to study physiological changes associated with emotional, cognitive, and autonomic regulatory processes.

HRV enables the characterization of cardiac dynamics through R-peak detection and the derivation of RR intervals, providing physiologically relevant information about autonomic modulation. Recent ECG- and HRV-based studies have shown that features derived from this signal can provide valuable information for the analysis of affective states and for a better physiological interpretation of cardiac responses [6]. Due to its non-invasive nature and well-established physiological basis, HRV has been widely used as a biomarker in the quantitative assessment of emotional and cognitive states. In addition, standardized methodologies for HRV analysis have reinforced its validity as a physiological indicator in psychophysiological studies [7,8].

HRV can be analyzed in both the time and frequency domains. In particular, spectral analysis makes it possible to study the distribution of signal energy across physiologically relevant bands, among which the low-frequency (LF, 0.04–0.15 Hz) and high-frequency (HF, 0.15–0.40 Hz) bands are especially important. These bands have been associated with different mechanisms of autonomic modulation and constitute a useful physiological reference for interpreting responses to different stimuli [4,7]. In this sense, variations in emotional states have been associated with changes in HRV spectral components, reflecting ANS modulation during affective responses [5,8]. Likewise, emotional responses are commonly described using frameworks such as the valence–arousal–dominance (VAD) model, which provides a multidimensional representation of affective states [9,10]. The integration of HRV analysis with this type of framework has supported the development of more robust approaches for physiological characterization in emotional contexts [11,12]. In the present work, however, emotional information is not used for classification purposes, but rather as an experimental context to study how autonomic modulation reflected in HRV varies during exposure to emotional stimuli.

Several approaches have been proposed for HRV spectral analysis. Traditional methods, such as the Fast Fourier Transform (FFT), provide frequency-domain representations but have limited spectral resolution when applied to short-duration or non-stationary signals [13,14]. Advanced spectral and time–frequency analysis techniques have been widely employed to characterize complex physiological dynamics, particularly under non-stationary conditions such as those commonly observed during emotional responses [15]. To address these limitations, time–frequency techniques such as the Continuous Wavelet Transform (CWT) have been employed, allowing non-stationary signals to be analyzed with improved temporal localization [16,17]. However, these methods depend on the appropriate selection of basis functions and may increase the complexity of spectral interpretation.

As an alternative, high-resolution spectral estimation methods have been explored. Among them, the Multiple Signal Classification (MUSIC) algorithm has shown favorable performance for identifying dominant components even in short recordings or under low signal-to-noise ratio conditions [14,18]. This capability makes it an attractive alternative for HRV analysis, where spectral components are often narrowband and difficult to resolve [19,20]. Subspace-based high-resolution spectral estimation techniques, including the MUSIC algorithm, have been applied to HRV analysis to improve frequency estimation from short-duration and noisy recordings [21].

Nevertheless, the performance of MUSIC strongly depends on the selection of the model order parameter *p*, which defines the dimension of the signal subspace [14]. An inappropriate choice of *p* may alter the localization of the dominant frequency, generate spurious spectral peaks, or favor components with limited physiological relevance. In practice, the selection of *p* is often empirical, and its effect on dominant frequency estimation may vary depending on the characteristics of the analyzed signal and the adopted configuration [19]. Consequently, the influence of model order on the spectral estimation of HRV signals, particularly under emotional stimulation, remains insufficiently explored. This limitation is especially relevant because the physiological interpretation of the dominant frequency directly depends on its stability and its consistency with the expected autonomic bands.

Based on the above, the aim of this work is to analyze the influence of the MUSIC model order on dominant frequency estimation in HRV signals obtained from ECG recordings under emotional stimulation. To this end, a spectral characterization methodology is proposed and applied to the DREAMER database, which integrates physiological recordings together with self-assessment emotional ratings [9]. Within this methodology, the consistency of the dominant frequency with the expected LF and HF bands is used as a physiological reference criterion. From this perspective, the dominant frequency of HRV can be employed as an indirect biomarker of autonomic modulation under emotional stimuli, according to its spectral localization within the LF and HF bands. In this sense, the main contribution of the study does not lie in the development of an emotion recognition system, but rather in systematically evaluating the effect of *p* on the stability, physiological coherence, and interpretability of the dominant frequency estimated by MUSIC, with the aim of strengthening its usefulness for HRV spectral characterization and for the analysis of autonomic responses under emotional stimulation.

## 2. Materials and Methods

### 2.1. DREAMER Database and Emotional Context

The present study was conducted using the DREAMER database, a publicly available multimodal dataset developed for emotion analysis from physiological signals acquired using low-cost wireless devices [9]. DREAMER includes electroencephalographic (EEG) and electrocardiographic (ECG) recordings obtained from participants exposed to affective audiovisual stimuli, together with post-stimulus self-assessments in terms of valence, arousal, and dominance (VAD).

The original DREAMER database contains valid recordings from 23 healthy participants after excluding two subjects whose data were incomplete due to technical issues during acquisition. Each participant was exposed to 18 video clips selected to elicit emotional responses associated with nine target affective states, namely, calmness, surprise, amusement, fear, excitement, disgust, happiness, anger, and sadness, with two clips assigned to each category.

For each stimulus, participants provided self-assessment scores on a five-point scale for valence, arousal, and dominance. These ratings were used as contextual information for the interpretation of HRV responses and for defining the a priori spectral tendency associated with each stimulus [9,10].

The emotional context of the DREAMER stimuli was interpreted according to the circumplex model of affect, which represents emotions within a two-dimensional space defined by valence and arousal [10]. Within this framework, valence describes the degree of pleasantness or unpleasantness associated with an emotional experience, whereas arousal reflects its level of activation or intensity. In this work, the circumplex model was used as a conceptual reference to contextualize the affective nature of the audiovisual stimuli and to support the physiological interpretation of HRV responses under emotional stimulation.

**Figure 1:**
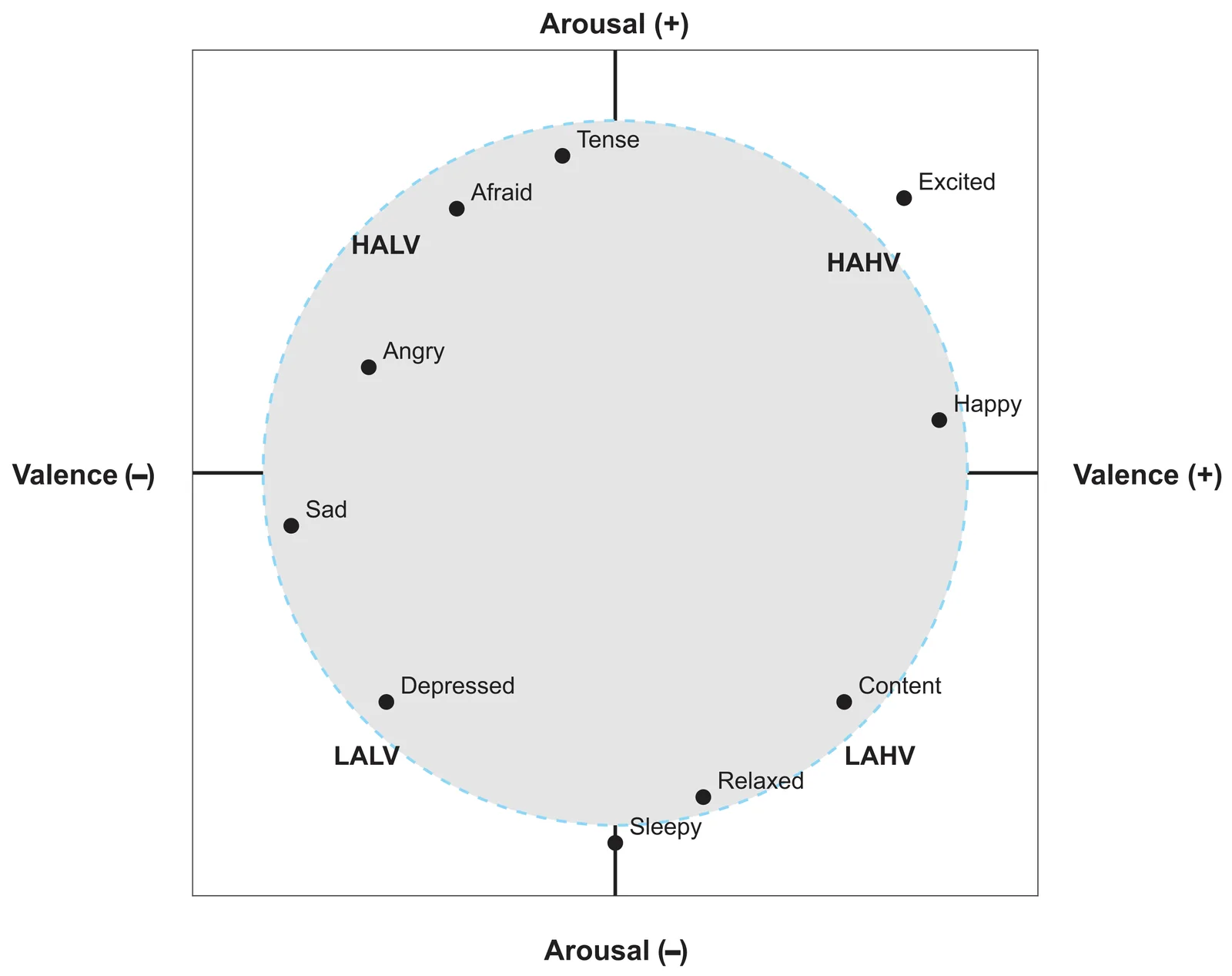
Circumplex model of affect based on Russell’s framework [10], illustrating the valence–arousal space and the distribution of representative emotional states.

Based on this representation, Figure 1 illustrates the valence–arousal space used as a conceptual reference to contextualize the emotional stimuli considered in this study.

The emotional context of each DREAMER stimulus was characterized using the valence–arousal–dominance (VAD) ratings reported in the original database. The valence and arousal dimensions were used to represent the stimuli within the circumplex framework.

For the valence–arousal representation, the midpoint of the DREAMER1–5 rating scale (3.0) was used as the reference value for defining high and low levels. Valence ratings above 3.0 were classified as high valence, whereas ratings below 3.0 were classified as low valence. Similarly, arousal ratings above 3.0 were classified as high arousal, while ratings below 3.0 were classified as low arousal. Ratings equal to 3.0 were considered neutral and treated as boundary cases within the classification scheme.

The quadrant assignment was determined exclusively from these numerical valence and arousal thresholds. The subsequent LF/HF assignment was a separate stimulus-level hypothesis and was therefore not defined solely by quadrant membership. Instead, the expected spectral tendency was established from the emotional context of each stimulus and the psychophysiological patterns reported in the literature. In general, stimuli associated with lower arousal or calmer emotional contexts were assigned an expected HF tendency, whereas stimuli characterized by higher arousal or stronger autonomic reactivity were assigned an expected LF tendency. These assignments were treated as descriptive, a priori spectral hypotheses rather than deterministic physiological rules.

The expected LF/HF assignment in Table 1 represents an a priori spectral hypothesis derived from the arousal ratings of the DREAMER stimuli. It is not intended to imply a deterministic relationship between emotional state and HRV spectral band, nor to identify LF or HF with a specific branch of autonomic activity. The expected band was used only as an independent reference for evaluating the dominant frequency estimates obtained after spectral analysis.

**Figure 2:**
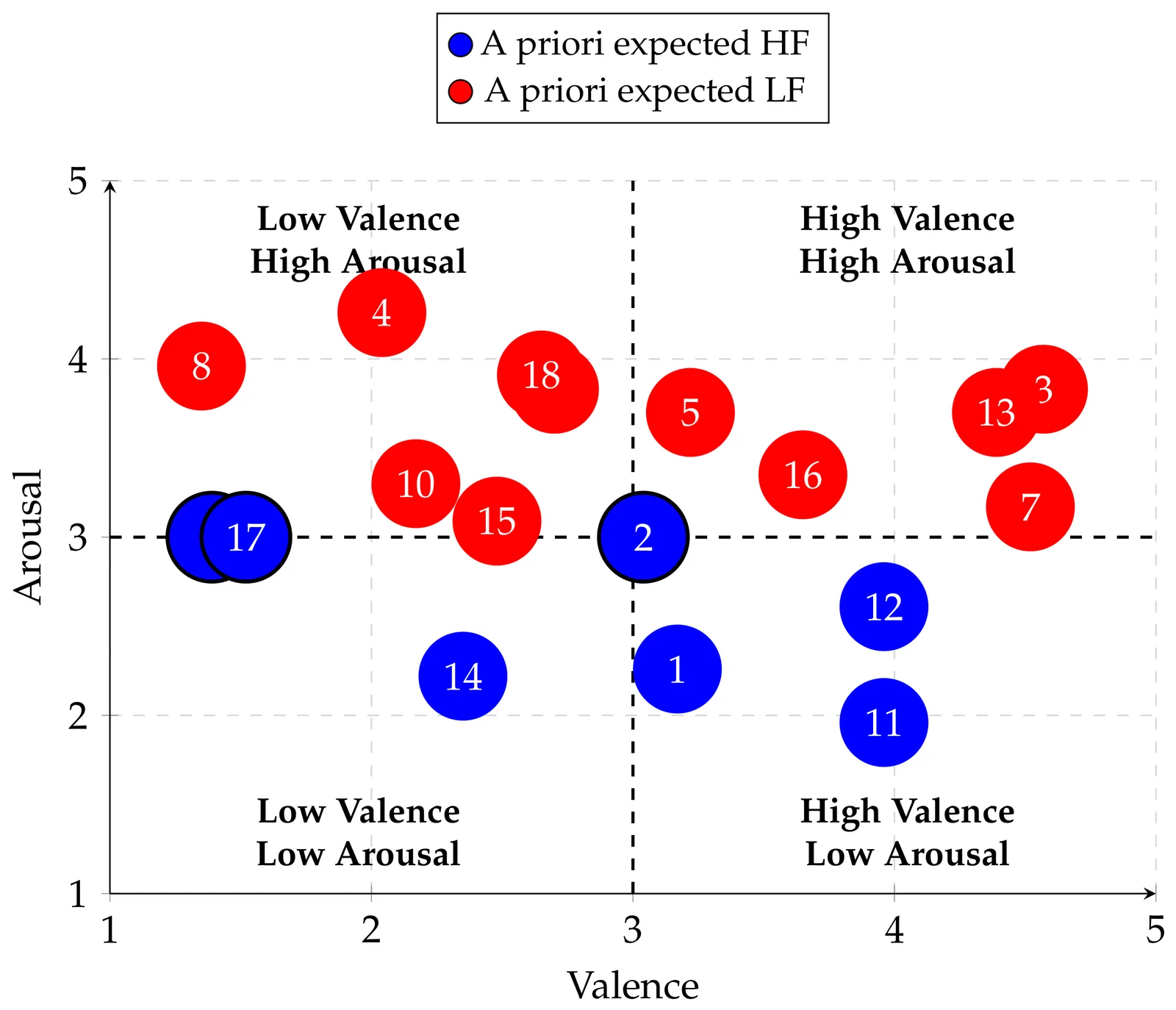
Valence–arousal representation of the DREAMER stimuli. Colors indicate the a priori expected LF/HF tendency. Dashed lines at 3.0 define the high/low thresholds. Numbers identify the corresponding stimuli listed in Table 1; some labels may be partially or completely obscured by overlapping markers. Stimuli 2, 9, and 17 are boundary cases. Assignments are descriptive hypotheses, not direct measures of autonomic activity.

To complement the stimulus-specific mapping summarized in Table 1, Figure 2 provides a visual representation of the DREAMER audiovisual stimuli in the valence–arousal plane. In this representation, each stimulus is positioned according to its mean affective ratings and color-coded according to its expected dominant HRV spectral tendency, allowing the distribution of HF- and LF-related conditions to be visualized within the emotional space of the database.

The LF and HF assignments shown in Figure 2 were used as a priori spectral hypotheses for the subsequent HRV analysis. The LF range (0.04–0.15 Hz) and HF range (0.15–0.40 Hz) are conventional frequency regions used in HRV analysis. Physiologically, these frequency ranges reflect different components of cardiovascular autonomic modulation; however, their interpretation is not specific to a single autonomic branch. In particular, LF power should not be interpreted as a direct measure of sympathetic activity, whereas HF power is commonly associated with respiratory-related vagal modulation. Accordingly, in the present study, the LF/HF assignment was used only to define an a priori frequency-domain hypothesis for each stimulus, rather than as a deterministic classification of sympathetic or parasympathetic activation.

The stimulus-specific expected HRV spectral tendencies presented in Table 1 were subsequently used as a priori reference categories for evaluating the dominant frequency estimates. Because these categories were defined exclusively from the DREAMER arousal ratings, they were independent of the spectral estimates obtained from the ECG recordings.

The physiological interpretation of the LF and HF ranges is discussed with appropriate caution in Section 2.5. In particular, the spectral bands are treated as frequency-domain descriptors rather than as direct measures of sympathetic or parasympathetic cardiac activity.

### 2.2. ECG Signals and HRV-Based Methodological Framework

In this work, only the ECG recordings from the DREAMER database were analyzed. According to the original acquisition protocol, ECG signals were recorded at a sampling frequency of 256 Hz using a wireless SHIMMER sensor, employing the right arm–left leg (RA–LL) lead configuration for subsequent analysis [9]. ECG-derived heart rate variability (HRV) was selected as the physiological signal of interest because it constitutes a non-invasive indicator of autonomic nervous system modulation and has been widely used in psychophysiological studies related to emotional processing [5,7,8].

For the purposes of this study, the complete set of ECG recordings corresponding to the 18 emotional stimuli and the 23 valid participants in the database was considered, resulting in a total of 414 signals analyzed for HRV spectral characterization.

To analyze autonomic modulation under emotional stimulation from a physiological perspective, this work proposes a methodology based on electrocardiographic signal processing and HRV analysis. In particular, ECG signals were processed to derive HRV signals, which provide a non-invasive representation of autonomic nervous system dynamics associated with emotional responses.

**Figure 3:**
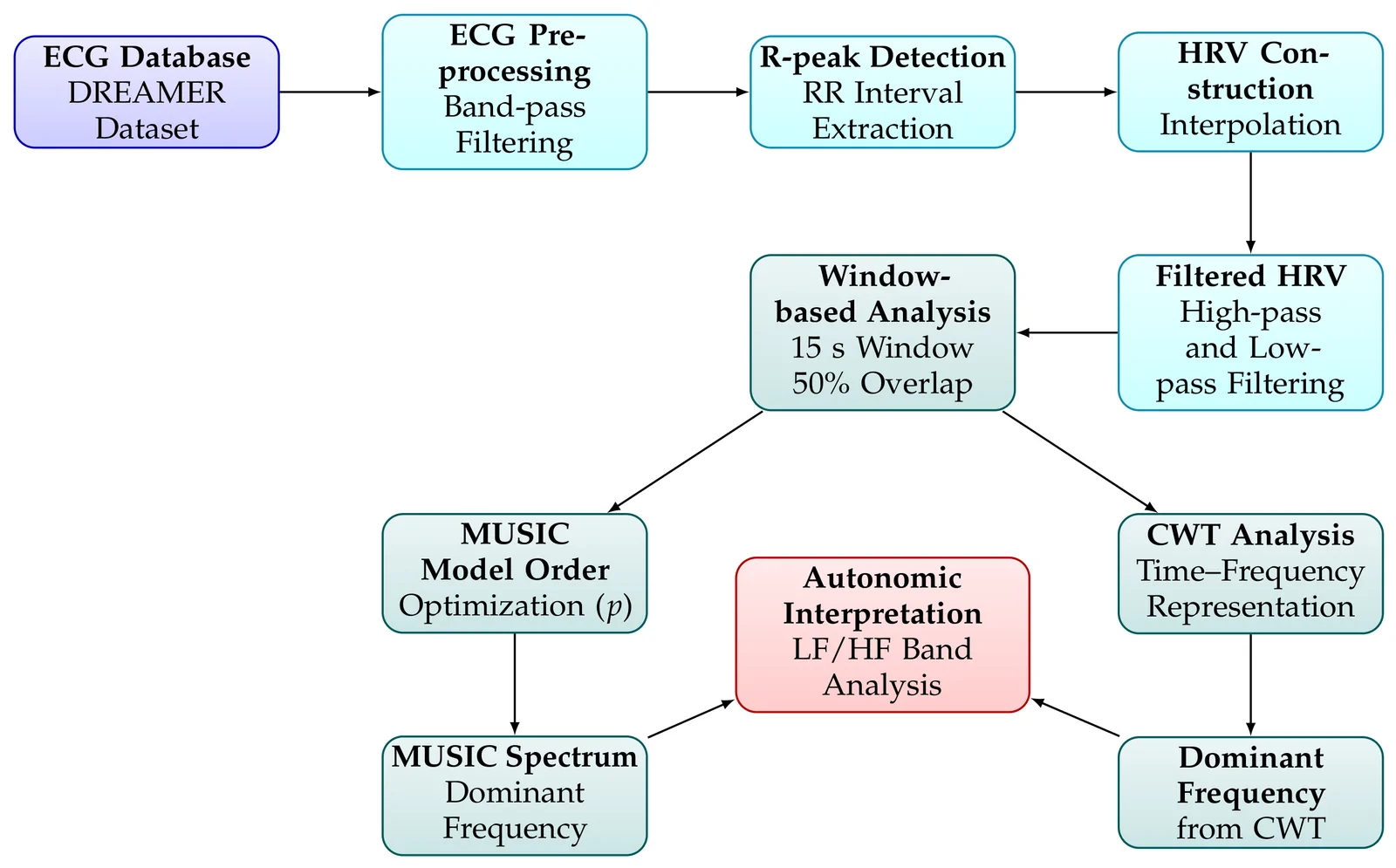
Block diagram of the proposed HRV analysis methodology using ECG signals from the DREAMER database. The framework includes ECG preprocessing, R-peak detection, HRV interpolation, filtering of the interpolated HRV signal, window-based spectral analysis, MUSIC model order optimization, CWT analysis, and autonomic interpretation based on LF/HF band analysis.

The proposed methodology consists of several stages, including ECG signal preprocessing, RR interval extraction, HRV signal construction, high-resolution spectral analysis using the Multiple Signal Classification (MUSIC) algorithm, model order optimization, and complementary time–frequency analysis through the Continuous Wavelet Transform (CWT). The resulting dominant frequency estimates were subsequently interpreted according to their spectral location within the LF and HF bands in order to characterize autonomic modulation under emotional stimulation, as illustrated in Figure 3.

### 2.3. ECG Preprocessing and HRV Signal Construction

The ECG signals from the DREAMER database were preprocessed to obtain the RR-interval series required for subsequent HRV spectral analysis. The DREAMER ECG recordings were processed at their original sampling frequency of 256 Hz. For each audiovisual stimulus, only the final 60 s of the recording were used for the principal stimulus-level analysis, following the standard stimulus-level analysis convention adopted in this study.

The ECG signal was band-pass filtered using a fifth-order Butterworth filter with cutoff frequencies of 0.5 and 40 Hz. This filtering stage was used to attenuate baseline fluctuations and high-frequency noise while preserving the main ECG components required for R-peak detection.

R-peaks were detected using the MATLAB R2024 findpeaks function with an amplitude threshold defined as the signal mean plus 0.5 standard deviations and a minimum inter-peak distance of 0.60 s. The detected RR intervals were then subjected to the quality-control procedure described below. No separate external validation of the R-peak detector was performed on the DREAMER recordings.

The resulting RR intervals underwent physiological and consistency-based quality control. Intervals outside the 0.30–2.00 s physiological range or deviating by more than 20% from the median RR interval were identified as potentially abnormal or artifactual. These intervals were corrected using PCHIP interpolation before subsequent HRV analysis. Across the 414 participant–stimulus recordings, 28,053 RR intervals were obtained. Of these, 504 intervals (1.80%) were flagged and corrected, corresponding to 122 recordings (29.47%) with at least one corrected interval. No recording was excluded because of insufficient R-peak detection or inadequate RR-interval quality.

Because the RR intervals are unevenly spaced in time, the corrected RR series was resampled at 8 Hz using piecewise cubic Hermite interpolation (PCHIP). No additional detrending was applied before filtering. The resulting uniformly sampled HRV signal was then processed using a second-order Butterworth band-pass filter with cutoff frequencies of 0.03 and 0.40 Hz. This range was selected to retain the frequency components considered in the subsequent LF/HF spectral characterization.

For the MUSIC analysis, the final 60 s HRV segment of each participant–stimulus recording was used. The MUSIC pseudo-spectrum was computed using $n_{\mathrm{fft}}=2048$ and a 21×21 Toeplitz autocorrelation/covariance matrix. Forward–backward averaging was not applied in the covariance estimation. The candidate model order range was defined consistently as p∈{2, 3,…,10}, and this same candidate range was used throughout the revised analysis.

### 2.4. Spectral Analysis of HRV Using MUSIC

Once the HRV signal was obtained in uniformly sampled form, spectral analysis was performed in order to identify its dominant oscillatory components and evaluate their distribution within physiologically relevant frequency bands. Since HRV reflects the temporal modulation of cardiac activity by the autonomic nervous system, its spectral characterization provides a useful framework for analyzing autonomic responses under emotional stimulation.

To estimate the dominant frequency of HRV with high spectral resolution, the Multiple Signal Classification (MUSIC) algorithm was employed. MUSIC is a subspace-based spectral estimation method that decomposes the covariance structure of the signal into signal and noise subspaces, allowing narrowband spectral components to be identified with greater resolution than conventional Fourier-based methods, particularly in short data records [14,18].

In this study, the MUSIC pseudo-spectrum was computed from the interpolated and filtered HRV signal in order to identify the dominant spectral component associated with autonomic modulation. The dominant frequency was defined as the frequency corresponding to the maximum value of the pseudo-spectrum within the analysis range, and its spectral location was subsequently evaluated with respect to the low-frequency (LF) and high-frequency (HF) bands.

Because the performance of MUSIC depends strongly on the selected model order, the spectral analysis was not limited to a fixed configuration. Instead, the influence of the model order parameter on dominant frequency estimation was explicitly examined as part of the proposed methodology, with the aim of evaluating its effect on spectral stability and physiological interpretability.

Let $R_{x}$ denote the autocorrelation matrix of the observed HRV signal. Through eigenvalue decomposition, $R_{x}$ can be expressed as follows:

(1) $$R_{x}=E_{s}\Lambda_{s}E_{s}^{H}+E_{n}\Lambda_{n}E_{n}^{H}$$

where $E_{s}$ and $E_{n}$ represent the signal and noise subspaces, respectively, while $\Lambda_{s}$ and $\Lambda_{n}$ are diagonal matrices containing the corresponding eigenvalues. A fundamental property of MUSIC is the orthogonality between the noise subspace and the signal steering vectors [14,18]. The MUSIC pseudo-spectrum was computed as follows:

(2) $$P_{\mathrm{MUSIC}}\left(f\right)=\frac{1}{a^{H}\left(f\right)E_{n}E_{n}^{H}a\left(f\right)}$$

where a(f) denotes the steering vector associated with frequency *f*. Peaks in the pseudo-spectrum correspond to the dominant frequency components of the HRV signal, enabling high-resolution frequency localization.

### 2.5. HRV Spectral Characterization and Physiological Interpretation

For spectral characterization, the dominant frequency estimated from the HRV signal was classified according to its location within the conventional low-frequency (LF) and high-frequency (HF) bands. The LF band was defined as 0.04–0.15 Hz, whereas the HF band was defined as 0.15–0.40 Hz  [7]. These ranges were used as spectral descriptors and not as direct indices of cardiac sympathetic or parasympathetic activity.

HRV spectral components reflect the combined influence of multiple cardiovascular regulatory mechanisms. The HF range is commonly associated with respiratory sinus arrhythmia and is strongly influenced by parasympathetic modulation, whereas variability within the LF range may arise from a combination of autonomic and baroreflex-related mechanisms. Importantly, LF power is not considered a specific index of cardiac sympathetic activity, and the LF/HF relationship should not be interpreted as a direct measure of sympathovagal balance [4,5,7,8].

An additional limitation concerns the absence of a directly measured respiratory signal in the DREAMER recordings. Because respiration strongly influences the HF component of HRV through respiratory sinus arrhythmia, changes in the location of the dominant HF frequency may reflect changes in respiratory rate and/or breathing pattern rather than changes in autonomic modulation alone. This consideration is particularly relevant during emotional stimulation, where respiration may change systematically between stimuli. Therefore, the present analysis does not interpret shifts in the HF peak as direct evidence of changes in parasympathetic activity or emotional autonomic activation. Similarly, a dominant frequency close to or below the LF/HF boundary cannot be attributed exclusively to sympathetic or parasympathetic mechanisms in the absence of respiratory measurements.

Accordingly, the LF/HF classification used in this study should be interpreted as a frequency-domain characterization of the HRV signal rather than as a respiratory-independent measure of autonomic or emotional activation. Direct assessment of respiration, including ECG-derived respiration or a simultaneously acquired respiratory channel, would be required to determine whether stimulus-related changes in the HF frequency are accompanied by corresponding changes in the respiratory rate.

In this study, LF/HF classification was treated as a frequency-domain characteristic of the HRV signal rather than as a direct measure of emotional state or autonomic activation. The emotional context provided by the DREAMER stimuli and their valence–arousal ratings was used to establish an a priori expected spectral tendency for each stimulus. This tendency was determined using the reproducible arousal threshold described above and specified whether the dominant spectral component was expected to occur within the LF or HF frequency range. The expected tendency was not used during MUSIC model order optimization and was evaluated only after the dominant frequency had been obtained.

Although the associations summarized in Table 2 provide a general psychophysiological reference for the affective and autonomic context of the stimuli, they are not interpreted as direct physiological labels for the LF or HF components observed in the present HRV analysis. The expected HRV spectral tendencies used throughout the analysis were defined by considering the general psychophysiological literature together with the valence–arousal–dominance ratings associated with each DREAMER stimulus. These tendencies were therefore treated as a priori spectral hypotheses rather than deterministic assignments of sympathetic or parasympathetic activity.

### 2.6. Model Order Optimization Strategy

A critical aspect in the application of the MUSIC algorithm is the selection of the model order parameter *p*, since it determines the dimension of the signal subspace and directly influences the spectral resolution and stability of the pseudo-spectrum. In HRV analysis, an inappropriate choice of *p* may produce spurious spectral peaks, unstable dominant frequency estimates, or an inadequate representation of the underlying spectral structure.

In the revised methodology, model order selection was formulated as a signal-driven procedure in order to avoid circularity between the optimization criterion and the subsequent physiological evaluation. Therefore, information related to the expected LF or HF band, emotional labels, valence, arousal, dominance, or any stimulus-specific physiological assignment was not used to select the model order. These variables were reserved exclusively for the post-selection interpretation and evaluation of the resulting dominant frequency.

The candidate model orders considered in this study were p∈{2, 3,…,10}. For each candidate order, the MUSIC pseudo-spectrum and complementary signal-derived criteria were calculated from the preprocessed HRV signal. The resulting measures were used to characterize the spectral and signal-subspace behavior of each candidate model order independently of the emotional condition.

#### 2.6.1. Signal-Driven Model Order Selection

The revised model order selection strategy was designed to remove the circularity identified in the original formulation. The selection of the MUSIC model order is performed exclusively from characteristics derived from the analyzed HRV signal and its estimated signal subspace. No expected LF/HF assignment, emotional label, valence, arousal, dominance, or other stimulus-specific physiological information is used during this selection.

For each HRV recording, all candidate model orders p∈{2,…,10} were evaluated. For each candidate order, the MUSIC pseudo-spectrum was computed from the preprocessed HRV signal, and the dominant frequency was obtained as

(3) $$f_{p}=\arg\max_{f}S_{p}\left(f\right),$$

where $S_{p}\left(f\right)$ denotes the MUSIC pseudo-spectrum obtained with model order *p*. The dominant frequency was used to characterize the spectral behavior of each candidate order but was not compared with an expected physiological band during model order selection.

In parallel, four signal-derived model order criteria were calculated: Akaike’s Information Criterion (AIC), Minimum Description Length (MDL), ESTER, and an eigenvalue-gap criterion. A covariance matrix of dimension M=21 was used to obtain the eigenvalues and eigenvectors required for these calculations. The eigengap was calculated from consecutive eigenvalues according to

(4) $$G\left(p\right)=\frac{\lambda_{p}}{\lambda_{p+1}},$$

where $\lambda_{p}$ and $\lambda_{p+1}$ denote consecutive ordered eigenvalues of the estimated covariance matrix. The logarithmic form logG(p) was used in the composite criterion.

The ESTER measure was obtained from the shift-invariance relationship of the estimated signal subspace. Let $U_{p}$ denote the first *p* eigenvectors of the covariance matrix. The matrix was partitioned into two overlapping submatrices $U_{1}$ and $U_{2}$, and the least-squares shift operator was estimated as

(5) $$\Psi_{p}=U_{1}^{†}U_{2}.$$

The corresponding shift-invariance error was calculated as

(6) $$E_{p}=U_{2}-U_{1}\Psi_{p},$$

and the ESTER measure was defined as

(7) $$\mathrm{ESTER}\left(p\right)=\frac{1}{∥E_{p}{∥}_{2}^{2}+\epsilon },$$

where ϵ is a small positive constant used to avoid numerical division by zero.

Because AIC, MDL, ESTER, and eigengap have different numerical scales, each criterion was normalized across the candidate model order range using min–max normalization:

(8) $$\tilde{C}\left(p\right)=\frac{C(p)-\min(C)}{\max(C)-\min(C)}.$$

For AIC and MDL, lower values are preferred and therefore their normalized values were used directly. For ESTER and eigengap, larger values indicate more favorable signal-subspace configurations; consequently, their normalized costs were defined as

(9) $$C_{\mathrm{ESTER}}\left(p\right)=1-\tilde{\mathrm{ESTER}}\left(p\right),$$

and

(10) $$C_{\mathrm{Gap}}\left(p\right)=1-\tilde{\log G}\left(p\right).$$

A normalized model-complexity term was also introduced:

(11) $$C_{\mathrm{Complexity}}\left(p\right)=\frac{p-p_{\min}}{p_{\max}-p_{\min}},$$

where $p_{\min}=2$ and $p_{\max}=10$.

The proposed signal-driven composite criterion was then defined as

(12) $$\begin{array}{cc} J_{\mathrm{SD}}\left(p\right)= & w_{\mathrm{AIC}}\tilde{\mathrm{AIC}}\left(p\right)+w_{\mathrm{MDL}}\tilde{\mathrm{MDL}}\left(p\right)\\ \; & +w_{\mathrm{ESTER}}C_{\mathrm{ESTER}}\left(p\right)+w_{\mathrm{Gap}}C_{\mathrm{Gap}}\left(p\right)\\ \; & +w_{\mathrm{Complexity}}C_{\mathrm{Complexity}}\left(p\right). \end{array}$$

Equal weights were used in the principal analysis:

(13) $$w_{\mathrm{AIC}}=w_{\mathrm{MDL}}=w_{\mathrm{ESTER}}=w_{\mathrm{Gap}}=w_{\mathrm{Complexity}}=0.20.$$

Because the signal-derived criteria have different numerical scales, each criterion was normalized across the candidate model order range using min–max normalization. The normalized criteria were then combined into a dimensionless composite objective. For the principal analysis, equal weights of 0.20 were assigned to AIC, MDL, ESTER, eigengap, and model complexity. These weights were predefined and were not adjusted according to LF/HF agreement, stimulus identity, emotional category, valence, arousal, or dominance.

A predefined weight-sensitivity analysis was additionally performed to evaluate the dependence of the selected model order on the relative contribution of the signal-derived criteria. Alternative configurations emphasizing information-theoretic criteria, subspace criteria, eigengap, and model complexity were evaluated over the complete DREAMER dataset. No physiological or emotional information was used to define or tune these alternative configurations.

The optimized model order for each recording was defined as

(14) $$p^{*}=\arg\min_{p\in \{2,\ldots ,10\}}J_{\mathrm{SD}}\left(p\right).$$

The final MUSIC pseudo-spectrum and dominant frequency were subsequently computed using $p^{*}$. Only after this selection was completed was the resulting dominant frequency evaluated with respect to the predefined physiological LF and HF bands. Thus, LF/HF information was used exclusively as an independent evaluation outcome and was not part of the optimization criterion.

The complete signal-driven model order selection procedure is summarized in Algorithm 1. For each HRV recording, all candidate model orders p=2,…,10 are evaluated using information-theoretic, signal-subspace, eigengap, and model-complexity characteristics. The selected order $p^{*}$ is then used to obtain the final MUSIC pseudo-spectrum and dominant frequency estimate. Importantly, the algorithm does not use the expected LF/HF band or emotional information during model order selection.
**Algorithm 1** Signal-driven MUSIC model order selection for HRV spectral analysis **Notation:** x[n] denotes the preprocessed HRV signal, *p* the candidate MUSIC model order, $f_{s}$ the HRV interpolation sampling frequency, and nfft the number of frequency points used for pseudo-spectrum estimation.  1:**Input:** Preprocessed HRV signal x[n], candidate model orders p∈{2,…,10}, sampling frequency $f_{s}$, and frequency-grid parameters.  2:**for** each candidate model order *p* **do**  3:    Compute the MUSIC pseudo-spectrum $\left[S_{p}\left(f\right),f\right]\leftarrow \mathrm{pmusic}\left(x\left[n\right],p,nfft,f_{s}\right)$  4:    Extract signal-derived model order criteria, including information-theoretic and subspace-based measures.  5:    Normalize the signal-derived criteria.  6: Compute the signal driven criterion $J_{\mathrm{SD}}\left(p\right)$ without using LF/HF information or emotional labels.  7:    Extract the candidate dominant frequency $f_{p}=\arg\max_{f}S_{p}\left(f\right)$  8:**end for**  9:Select the optimized model order $p^{*}=\arg\min_{p}J_{\mathrm{SD}}\left(p\right)$10:Compute the final MUSIC pseudo-spectrum using $p^{*}$11:Extract the optimized dominant frequency $f_{\mathrm{dom}}=\arg\max_{f}S_{p^{*}}\left(f\right)$12:Independently evaluate $f_{\mathrm{dom}}$ with respect to the LF and HF physiological bands.13:**Output:** Optimized model order $p^{*}$, dominant frequency $f_{\mathrm{dom}}$, and post-selection LF/HF classification.

#### 2.6.2. Benchmark Model Order Criteria

To evaluate whether the proposed signal-driven strategy provided model order selections that were distinguishable from conventional approaches, several complementary model order criteria were evaluated. The comparison included a fixed reference configuration of p=4, AIC, MDL, ESTER, and eigengap-based selection, together with the proposed signal-driven composite criterion.

The fixed p=4 configuration was retained as an empirical reference because this value had been used in the original analysis of the manuscript. Importantly, this fixed configuration was not treated as the optimized solution; rather, it served as a baseline against which adaptive model order strategies could be compared.

For the information-theoretic approaches, AIC and MDL were evaluated as independent criteria for determining the model order from the statistical representation of the signal. ESTER and eigengap methods were included as complementary subspace-based approaches, since their selection mechanisms depend on the separation and structure of the estimated signal and noise subspaces.

All benchmark methods were evaluated over the same candidate range, p∈{2,3,…,10}, and using the same preprocessed HRV recordings. This common candidate range and common signal-processing pipeline allowed the resulting model order distributions to be compared without introducing differences attributable to preprocessing or data selection.

The comparison was performed across the complete DREAMER dataset, comprising the 23 valid participants and 18 audiovisual stimuli. Thus, the benchmark analysis considered the complete set of 414 participant–stimulus recordings rather than relying exclusively on representative examples.

#### 2.6.3. Independent Physiological Evaluation

After the model order had been selected, the final MUSIC pseudo-spectrum was calculated using $p^{*}$, and the corresponding dominant frequency was extracted. Only at this stage was the dominant frequency evaluated with respect to the physiological HRV bands.

The LF and HF bands were defined as LF=0.04–0.15Hz, and HF=0.15–0.40Hz, following the HRV frequency-domain framework described in Section 2.5. Accordingly, the complete analytical sequence was $\mathrm{HRV}\to p^{*}\to f_{\mathrm{dom}}\to \mathrm{LF}/\mathrm{HF}\;\mathrm{evaluation}$.

This separation ensures that physiological band information is an outcome of the model order selection process rather than an input to it. Therefore, agreement between the resulting dominant frequency and the expected physiological band can be interpreted as an independent evaluation of the spectral characterization rather than as evidence that the band information was used to select *p*.

The emotional information available in DREAMER, including the valence–arousal–dominance ratings and the stimulus-specific classification described in Table 1, was consequently used only for post-selection interpretation and descriptive analysis. It was not included in $J_{\mathrm{SD}}\left(p\right)$ and did not influence the determination of $p^{*}$.

#### 2.6.4. Window-Based Model Order Analysis

In addition to the recording-level model order selection, a window-based analysis was performed to examine the temporal behavior of the selected model order within individual HRV recordings. The interpolated HRV signal was divided into partially overlapping temporal windows, and the signal-driven model order procedure was independently applied to each segment.

For each temporal window *w*, the corresponding optimal order was defined as

(15) $$p^{*}\left(w\right)=\underset{p\in \{2,\ldots ,10\}}{\arg\min}\;J_{\mathrm{SD}}\left(p,w\right),$$

where $J_{\mathrm{SD}}\left(p,w\right)$ denotes the signal-driven criterion calculated for the *w*-th temporal segment.

The resulting sequence $p^{*}\left(w\right)$ was used to characterize temporal variations in the spectral structure of the HRV signal. A representative recording-level model order was obtained from the distribution of the window-level solutions, allowing the method to retain a single model order representation when required while preserving information about local temporal changes.

The implementation of the complete procedure was performed in MATLAB^®^. The same preprocessing, candidate-order range, and spectral analysis settings were maintained for all model order criteria and for all 414 participant–stimulus recordings. This common implementation allowed the effect of the model order selection strategy to be evaluated independently of the ECG preprocessing and HRV construction stages.

### 2.7. Statistical Analysis

Because the DREAMER dataset contains repeated measurements from the same participants, participant identity was treated as a random effect and emotional condition as a fixed effect in linear mixed-effects models [31]. Separate models were fitted for the selected model order $p^{*}$ and the dominant frequency $f_{\mathrm{dom}}$.

For each response, the model including emotional condition was compared with a corresponding null model using a likelihood-ratio test. Marginal and conditional $R^{2}$ values ($R_{m}^{2}$ and $R_{c}^{2}$) were calculated to quantify the variance explained by the fixed effects and by the complete model, respectively [32,33].

In addition, 95% confidence intervals for the mean $p^{*}$ and $f_{\mathrm{dom}}$ were estimated by participant-level bootstrap resampling [34]. For the within-subject baseline comparison, stimulus-level and corresponding baseline HRV estimates were compared within each participant using the paired Wilcoxon signed-rank test [35]. The corresponding paired effect size was quantified using Cohen’s $d_{z}$ [36].

For completeness, no pairwise post hoc comparisons were performed. Therefore, no multiplicity correction was applied. Statistical significance was evaluated using a two-sided threshold of p<0.05. Confidence intervals and effect sizes were considered together with the *p*-values.

## 3. Results

### 3.1. Example of ECG-to-HRV Processing and Spectral Analysis

This section presents the main experimental results obtained from the HRV analysis framework applied to the complete set of ECG recordings from the DREAMER database. Although all 18 emotional stimuli and the 23 valid participants were analyzed, some figures are presented using representative examples to facilitate the description of the processing pipeline and the interpretation of the spectral results. In particular, selected results corresponding to the calmness condition are included only as illustrative cases of the proposed methodology and should not be interpreted as the sole basis of the analysis.

**Figure 4:**
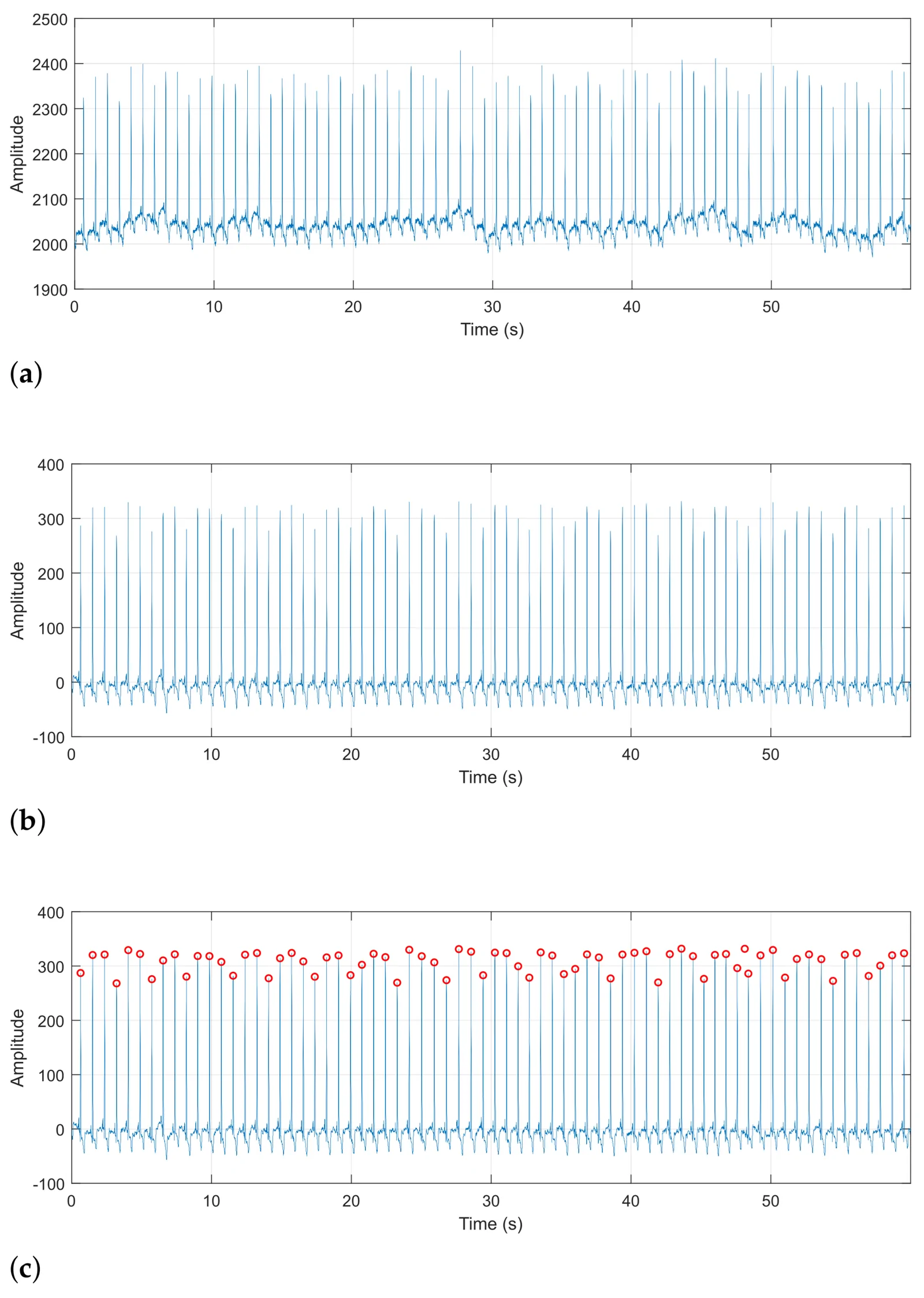
ECG preprocessing stages for Subject 9 under Stimulus 11 (calmness): raw ECG, band-pass filtered ECG, and detected R-peaks. The horizontal axis represents time (s), and the vertical axis represents ECG amplitude (mV). The recording segment shown corresponds to the analyzed 60 s stimulus interval. Red circles indicate the detected R-peaks. (**a**) Raw ECG signal for Subject 9 under Stimulus 11 (calmness). (**b**) Band-pass filtered ECG signal for Subject 9 under Stimulus 11 (calmness). (**c**) Detected R-peaks from the filtered ECG signal for Subject 9 under Stimulus 11 (calmness).

**Figure 5:**
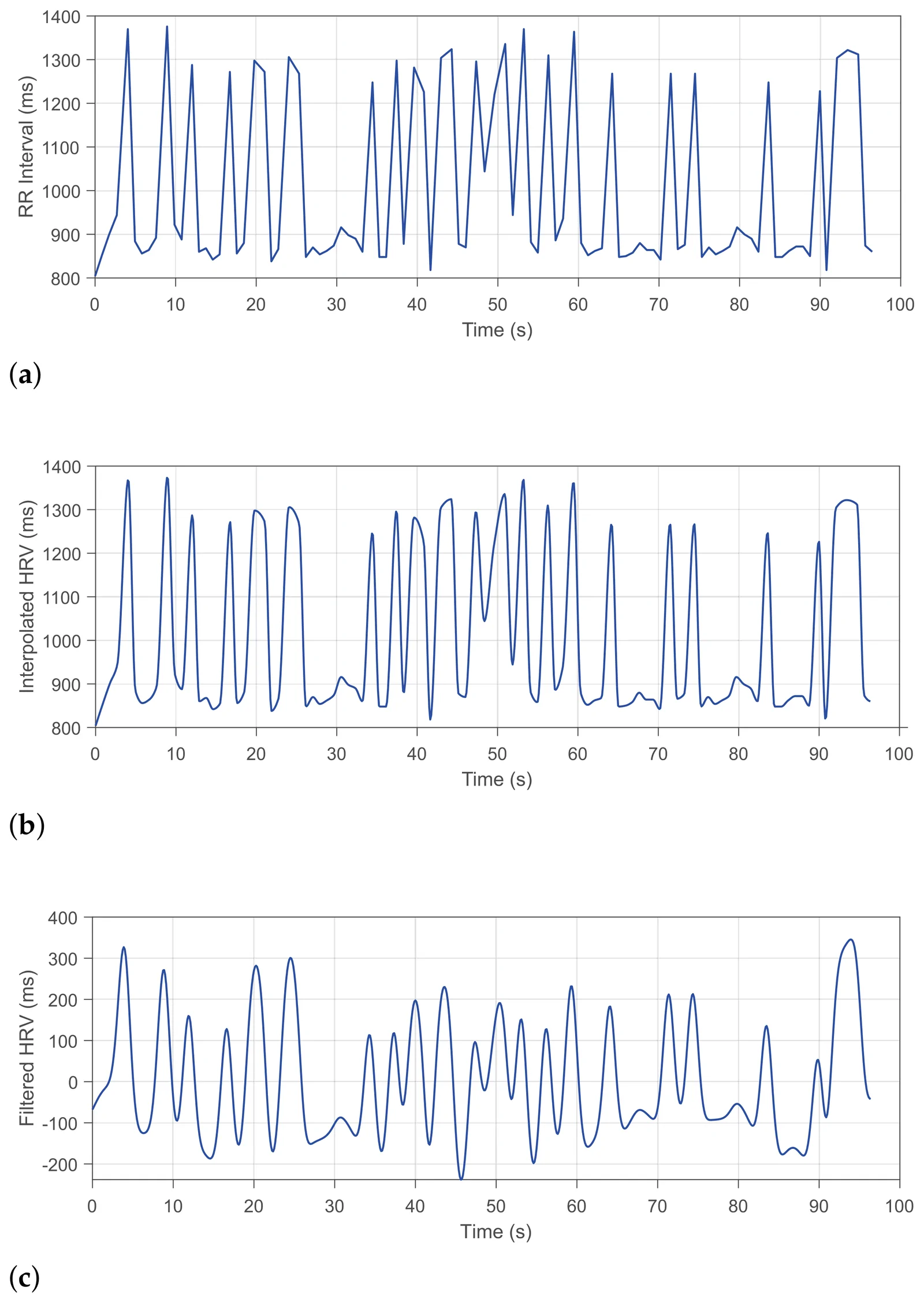
HRV generation and preprocessing stages for Subject 9 under Stimulus 11 (calmness): RR-derived HRV, PCHIP-interpolated HRV, and filtered interpolated HRV. The horizontal axis represents time (s), and the vertical axis represents RR interval (s). The interpolated HRV was sampled at 8 Hz, and the final signal was filtered in the 0.03–0.40 Hz range. The recording segment shown corresponds to the analyzed 60 s stimulus interval. (**a**) HRV signal obtained from RR intervals for Subject 9 under Stimulus 11 (calmness). (**b**) Interpolated HRV signal used for spectral analysis for Subject 9 under Stimulus 11 (calmness). (**c**) Filtered interpolated HRV signal used for spectral analysis for Subject 9 under Stimulus 11 (calmness).

Figure 4 and Figure 5 present a representative example of the ECG-to-HRV processing stages for one of the analyzed recordings. The example corresponds to Subject 9 under Stimulus 11 (calmness), and it is included only to illustrate the proposed processing sequence, which was applied to all recordings in the DREAMER database. Specifically, Figure 4 shows the ECG preprocessing stages, including band-pass filtering and R-peak detection, whereas Figure 5 presents the corresponding HRV signal obtained from the RR intervals, together with its interpolation and final conditioning prior to spectral analysis.

As observed in Figure 4, the filtering stage effectively attenuated baseline fluctuations and high-frequency noise while preserving the morphology of the QRS complexes, which facilitated reliable R-peak identification. In turn, Figure 5 illustrates the transformation from the irregular RR interval sequence to the interpolated and filtered HRV signal used for subsequent spectral analysis. These preprocessing stages provided the uniformly sampled and physiologically conditioned HRV signals required for the application of the MUSIC algorithm.

**Figure 6:**
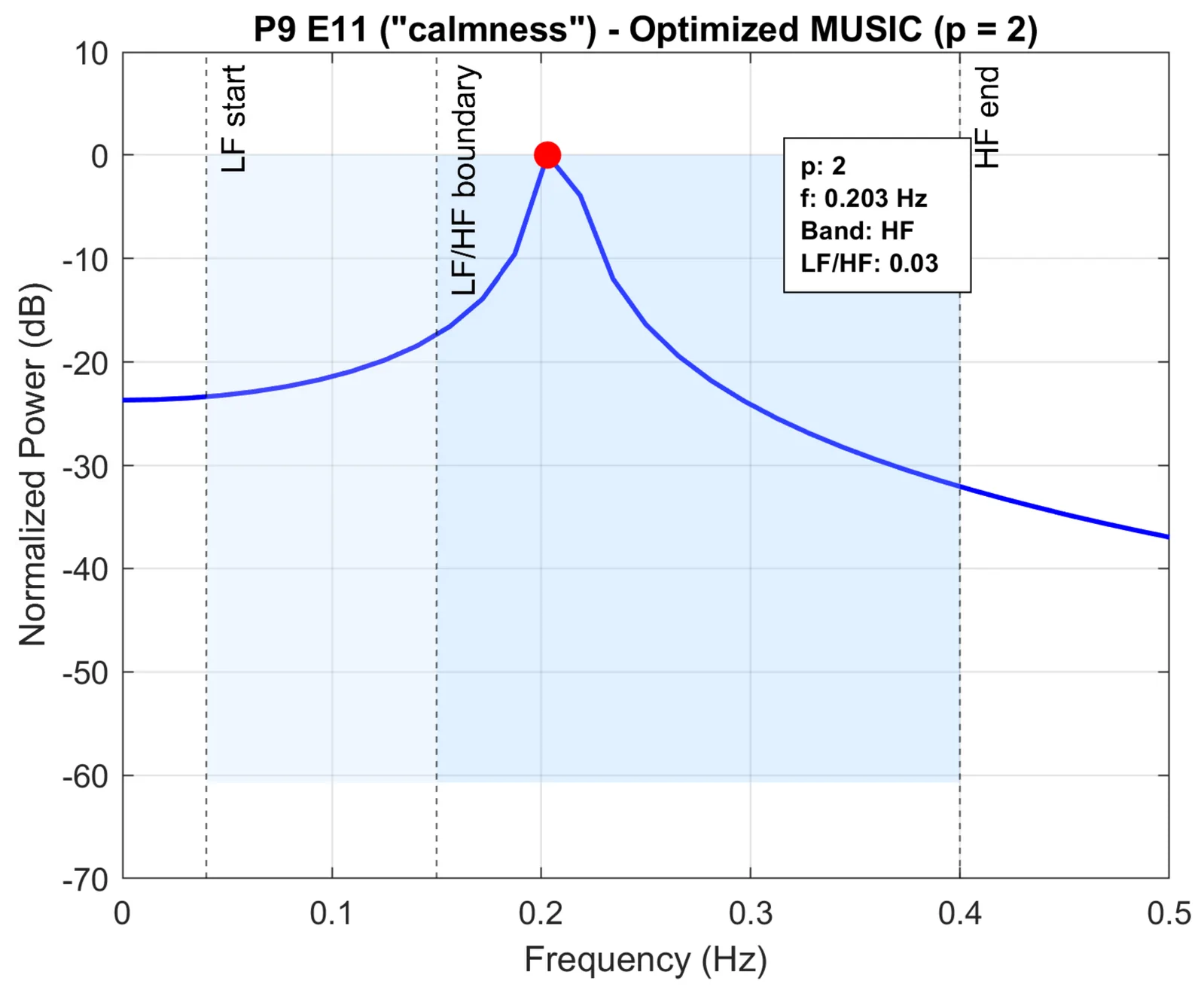
Normalized MUSIC pseudo-spectrum for Subject 9 under Stimulus 11 (calmness), obtained from the final 60 s HRV segment using the signal-driven model order selection procedure. The horizontal axis represents frequency (Hz) and the vertical axis represents the normalized MUSIC pseudo-spectrum. The LF and HF regions are indicated using the 0.04–0.15 Hz and 0.15–0.40 Hz frequency ranges, respectively, with the LF/HF boundary marked at 0.15 Hz.

The spectral behavior of the representative HRV signal was further evaluated using the optimized MUSIC configuration. Figure 6 shows the normalized MUSIC pseudo-spectrum obtained for Subject 9 under Stimulus 11 (calmness). The dominant frequency was identified within the HF band. In the revised analysis, the MUSIC pseudo-spectrum was used only to identify the dominant frequency and was not interpreted as a power spectral density. Therefore, no LF/HF power ratio was derived from the MUSIC pseudo-spectrum.

**Figure 7:**
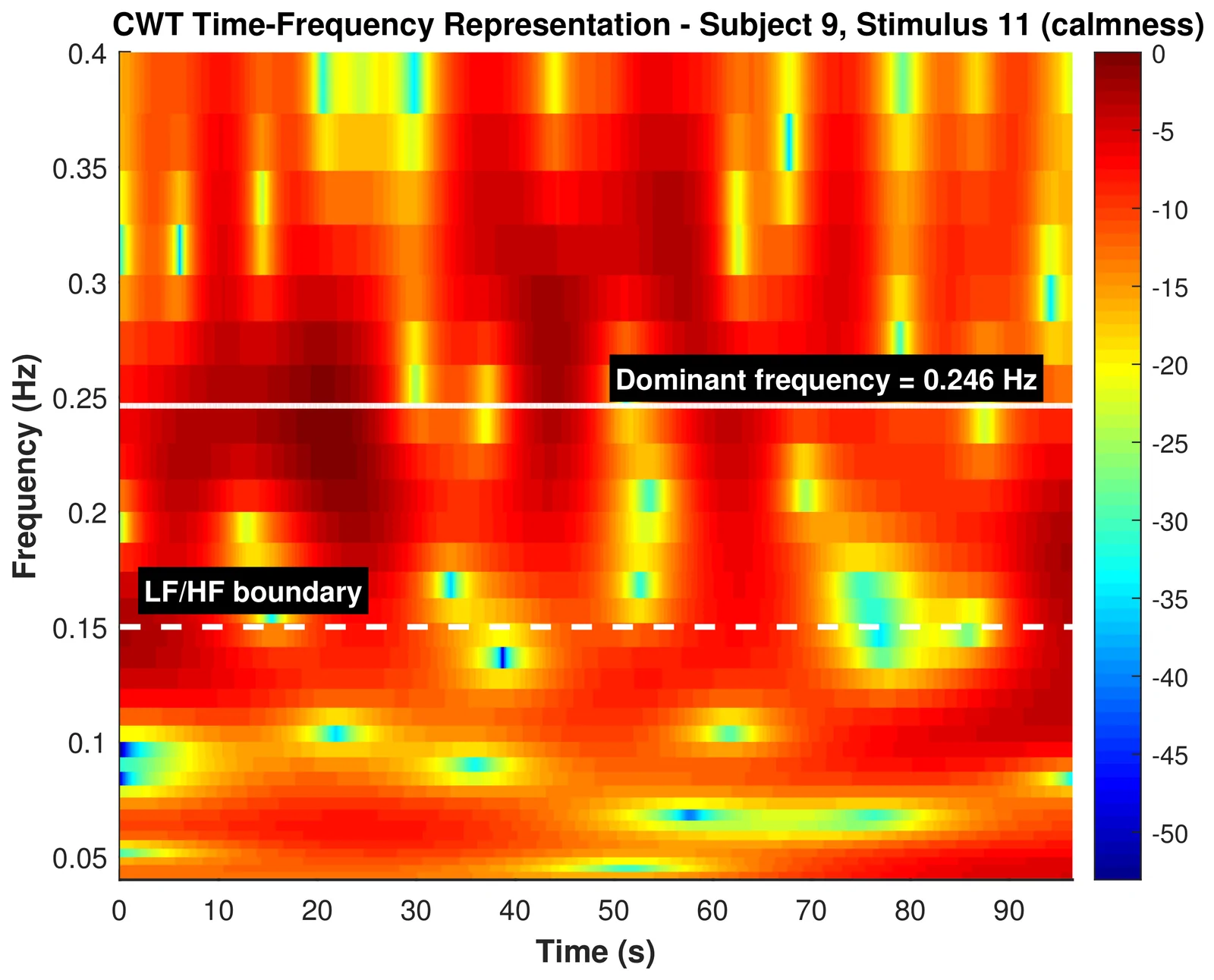
Time–frequency representation of the HRV signal for Subject 9 under Stimulus 11 (calmness) obtained using the Continuous Wavelet Transform (CWT). The horizontal axis represents time (s), and the vertical axis represents frequency (Hz), covering the analyzed 0.04–0.40 Hz frequency range. The dashed white line at 0.15 Hz indicates the LF/HF boundary, while the upper dashed line marks the dominant frequency estimated from the CWT representation. The color scale represents the normalized CWT magnitude.

To complement the spectral characterization obtained with the optimized MUSIC analysis, Figure 7 presents the time–frequency representation of the same HRV recording obtained with the Continuous Wavelet Transform (CWT). This complementary analysis was included to visualize how the spectral energy of the HRV signal is distributed over time and to contrast the dominant frequency localization obtained with MUSIC from a time–frequency perspective.

Figure 7 shows the HRV scalogram obtained with the Continuous Wavelet Transform (CWT) for Subject 9 under Stimulus 11 (calmness). In this representation, the spectral energy is mainly distributed within the high-frequency region, and the global dominant frequency estimated from the scalogram was 0.246 Hz. Since this value lies within the HF band, the result is physiologically consistent with the parasympathetic-related autonomic modulation expected for the calmness condition. In this work, the CWT was not used to optimize the model order, but rather as a complementary time–frequency reference to contrast the dominant frequency localization obtained from the optimized MUSIC analysis.

Taken together, the MUSIC and CWT representations obtained for the illustrative calmness case showed a dominant localization within the HF band, in agreement with the parasympathetic-related modulation expected for this emotional condition. While MUSIC enabled the identification of the dominant frequency from a high-resolution pseudo-spectrum, the CWT provided a complementary time–frequency reference to visualize the temporal distribution of HRV spectral energy. This example was included only for illustrative purposes to demonstrate the physiological interpretation of the spectral analysis; however, the complete evaluation was performed across the 414 signals corresponding to the 18 stimuli and the 23 valid participants from the DREAMER database.

### 3.2. Performance of Signal-Driven Model Order Selection

The revised signal-driven model order selection strategy was evaluated over the complete DREAMER dataset, comprising 23 participants and 18 audiovisual stimuli per participant. Thus, the analysis included 414 participant–stimulus recordings. For each recording, the candidate MUSIC model orders p∈{2,…,10} were evaluated using the same preprocessing, HRV construction, covariance estimation, and spectral analysis procedures.

Importantly, model order selection was performed independently of the physiological interpretation of the stimulus. No expected LF/HF assignment, emotional label, valence, arousal, or dominance information was used to determine the selected model order. These variables were considered only after the final model order and dominant frequency had been obtained.

To evaluate the behavior of the proposed strategy, its selected model orders were compared with those obtained using a fixed p=4 configuration, AIC, MDL, ESTER, and eigengap criteria. The fixed p=4 configuration was retained as the empirical reference because this value was used in the original analysis of the manuscript. All comparison methods were evaluated using the same candidate range and the same 414 HRV recordings.

Table 3 summarizes the resulting model order statistics and the corresponding mean dominant frequencies. The benchmark criteria produced substantially different model order distributions. AIC and MDL consistently selected the maximum candidate order, p=10, across the analyzed recordings. In contrast, ESTER and eigengap produced lower-order solutions, with mean values of 2.942 and 3.367, respectively. The proposed signal-driven criterion resulted in a mean model order of 3.659, a standard deviation of 0.490, and a median of 4, with selected orders ranging from p=3 to p=5.

These results demonstrate that different model order selection criteria lead to different model order distributions and that the selected order is not uniquely determined by the MUSIC algorithm itself. The proposed criterion does not simply reproduce the maximum candidate order selected by AIC and MDL, nor does it consistently select the lowest orders obtained with ESTER and eigengap. Instead, it produces a recording-dependent model order distribution with p=4 as the most frequently selected order.

The selected model order also affected the resulting dominant frequency estimates. The mean dominant frequency obtained using the proposed criterion was 0.1514 Hz, compared with 0.1168 Hz for the fixed p=4 configuration, 0.1048 Hz for both AIC and MDL, 0.1625 Hz for ESTER, and 0.1613 Hz for eigengap. These differences demonstrate that the choice of model order criterion can affect the resulting spectral characterization of HRV.

For the proposed equal-weight configuration, p=4 was selected in 64.49% of the recordings, whereas p=3 was selected in 34.78% and p=5 in 0.72%. Thus, 99.28% of the recordings were assigned either p=3 or p=4, while retaining recording-level adaptability. The complete distribution of the selected model orders is reported in Table 4.

**Figure 8:**
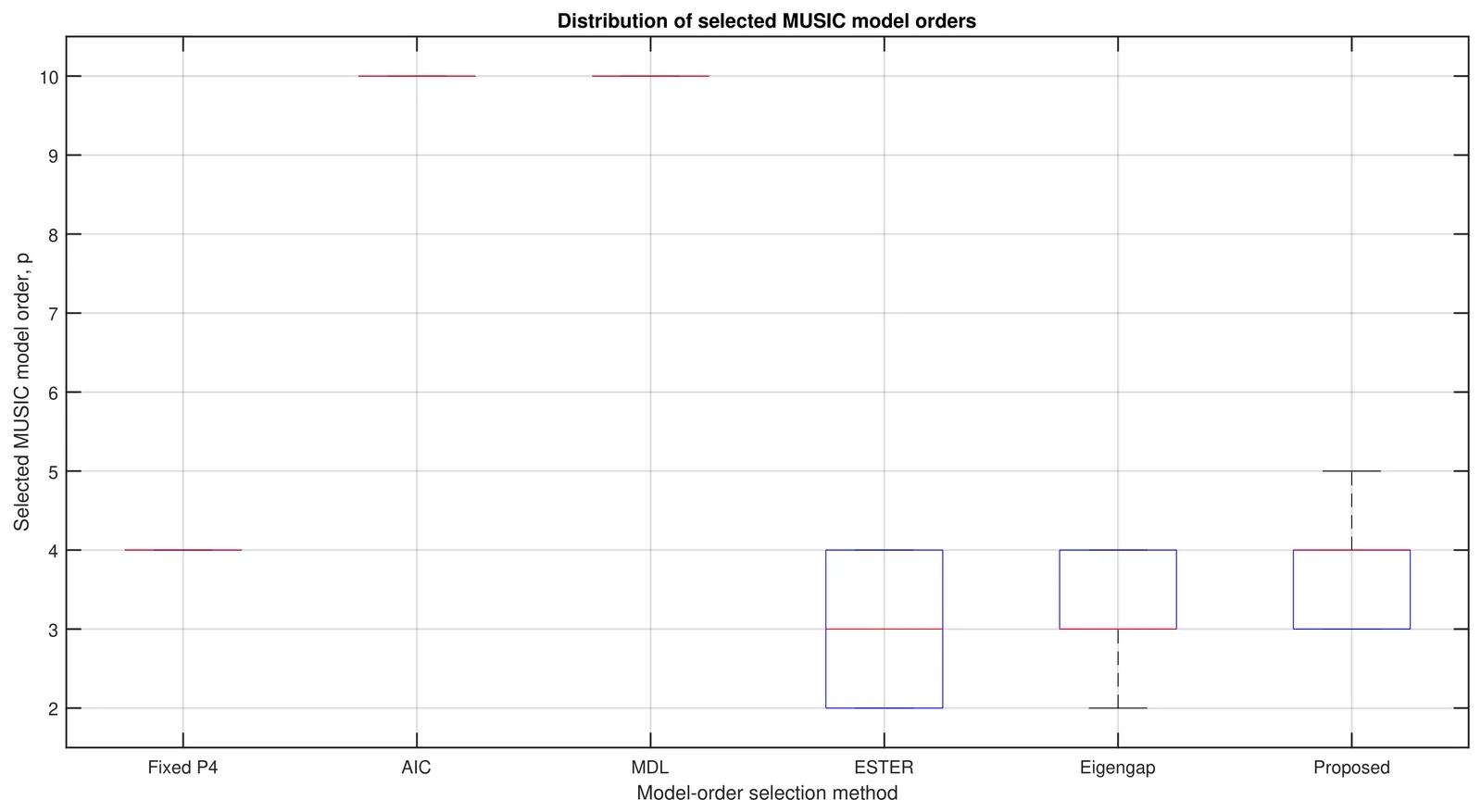
Distribution of the selected MUSIC model orders obtained with the fixed p=4, AIC, MDL, ESTER, eigengap, and proposed signal-driven criteria across the 414 DREAMER participant–stimulus recordings. AIC and MDL consistently selected the upper candidate order (p=10), whereas the proposed criterion selected recording-dependent orders within p=3–5. No physiological or emotional information was used during model order selection.

Figure 8 summarizes the distribution of the selected MUSIC model orders across the 414 DREAMER participant–stimulus recordings. The fixed p=4 configuration produced no variability in the selected order, as expected. AIC and MDL also converged systematically to the upper boundary of the candidate range, p=10, with zero variability across the dataset. In contrast, ESTER and eigengap selected lower model orders, with median values of p=3 and ranges of p=2–4. The proposed signal-driven criterion selected model orders between p=3 and p=5, with a median of p=4 and a standard deviation of 0.490.

These results indicate that the proposed criterion preserves the advantages of an adaptive model order strategy while avoiding systematic selection of either the maximum or minimum candidate orders. The selected order remains recording-dependent and is determined exclusively from the signal-derived characteristics used by the proposed criterion.

### 3.3. Spectral-Band Agreement Across Emotional Stimuli

After the signal-driven model order selection was completed, the resulting dominant frequency estimates were evaluated independently with respect to the expected LF/HF spectral tendency associated with each DREAMER stimulus. The expected band was defined a priori from the stimulus-level hypothesis and was not provided to the model order optimization procedure.

For each stimulus, the selected model orders and dominant frequencies were first summarized across the 23 participant recordings. Table 5 reports the mean selected model order, the mean dominant frequency, the percentage of recordings classified within the LF and HF ranges, and the corresponding agreement with the expected stimulus-level band.

The results show that the signal-driven procedure produced recording-level model orders that varied across stimuli, with mean values ranging from approximately p=3.48 to p=3.87. The corresponding mean dominant frequencies also varied across stimuli, ranging from 0.1199 to 0.1948 Hz. After model order selection, these dominant frequencies were evaluated with respect to the a priori expected LF/HF spectral tendency. The resulting agreement was not uniform across all stimuli.

Overall, 57.25% of the participant–stimulus recordings showed agreement between the estimated dominant frequency and the predefined spectral tendency. The stimulus-level agreement ranged from 26.09% to 82.61%. The highest agreement was observed for Disgust (stimulus 6, 82.61%), whereas the lowest agreement was obtained for Amusement (stimulus 12, 26.09%) and Calmness (stimulus 11, 34.78%). These differences indicate that the correspondence between the estimated dominant frequency and the a priori spectral tendency varied substantially across stimuli.

A further observation is that agreement was generally higher for stimuli assigned a priori to the LF range than for those assigned to the HF range. For the LF-assigned stimuli, agreement values ranged from 43.48% to 82.61%, whereas for the HF-assigned stimuli they ranged from 26.09% to 47.83%. This asymmetry indicates that the predefined spectral hypothesis was not equally supported across the two frequency ranges and should therefore be interpreted as an empirical spectral tendency rather than as a deterministic physiological relationship.

The observed agreement does not imply that a particular emotion is intrinsically associated with either the LF or HF band. The expected bands were defined a priori from the stimulus-level VAD characteristics of the DREAMER recordings and the corresponding spectral hypothesis. They were not provided to the MUSIC optimization procedure and were not used to select the model order. Consequently, the agreement values represent an independent post-selection comparison between the estimated dominant frequency and the predefined spectral tendency.

The 57.25% agreement should not be interpreted as an accuracy measure or as evidence of improvement over chance. The expected LF/HF designation represents an a priori stimulus-level spectral hypothesis rather than an independently measured physiological ground truth. Therefore, the reported percentages are used descriptively to characterize the correspondence between the estimated dominant frequency and the predefined spectral tendency. The model order was selected independently for each recording, and the expected LF/HF designation was not used during the MUSIC optimization.

### 3.4. Mixed-Effects Analysis of Model Order and Dominant Frequency

Because the dataset contains repeated measurements from the same participants across multiple emotional stimuli, a linear mixed-effects framework was used to evaluate whether the selected model order and dominant frequency varied across emotional conditions. Participant identity was included as a random effect, while emotional condition was included as a fixed effect. The analysis included 414 observations corresponding to 23 participants and 18 audiovisual stimuli.

For the selected model order $p^{*}$, the likelihood-ratio comparison between the model including emotional condition and the corresponding null model yielded $\chi^{2}\left(8\right)=18.99$, p=0.0149. The marginal and conditional coefficients of determination were $R_{m}^{2}=0.0321$ and $R_{c}^{2}=0.3178$, respectively. For the dominant frequency $f_{\mathrm{dom}}$, the corresponding likelihood-ratio comparison yielded $\chi^{2}\left(8\right)=18.87$, p=0.0155, with $R_{m}^{2}=0.0329$ and $R_{c}^{2}=0.2951$.

These results provide evidence of statistically detectable differences among emotional conditions for both the selected model order and dominant frequency when participant-level repeated measurements are accounted for. However, the relatively small marginal $R^{2}$ values indicate that the emotional condition explains only a small proportion of the total variance. Therefore, these results should not be interpreted as evidence of a strong emotion-dependent effect or as evidence that the proposed procedure improves spectral estimation.

To characterize the uncertainty of the stimulus-level estimates, participant-level bootstrap 95% confidence intervals were also calculated. These intervals are reported for the mean selected model order and dominant frequency for each emotional condition. No pairwise post hoc comparisons between emotional conditions were performed; therefore, no multiplicity correction was applied. The statistical analysis is consequently limited to the omnibus mixed-effects tests and the corresponding effect-size estimates.

### 3.5. Within-Subject Comparison with Baseline

To evaluate the influence of inter-individual variability in the dominant frequency estimates, the DREAMER baseline recordings were processed using the same procedure applied to the stimulus recordings. For each participant, the baseline dominant frequency was compared with the stimulus-derived estimates after averaging the 18 stimulus recordings within participant.

Across the 23 participants, the mean stimulus-minus-baseline difference in dominant frequency was 0.0215 Hz (95% CI: 0.0099–0.0330 Hz). The paired Wilcoxon test indicated a statistically detectable difference between baseline and stimulus conditions (p=0.0007), with a paired Cohen’s $d_{z}=0.80$.

These results provide a participant-aware reference for interpreting the absolute dominant frequency estimates. The baseline comparison was performed after the signal-driven MUSIC model order selection and was not used as an input to the optimization procedure. Therefore, it does not constitute a baseline-informed model order selection criterion. Instead, it provides an independent within-subject assessment of the change in dominant frequency associated with the stimulus recordings.

### 3.6. Comparison with Conventional Spectral Estimators

Table 6 provides a stimulus-level comparison of the dominant frequency estimates obtained with MUSIC, Welch, and the Lomb–Scargle periodogram. The comparison was performed only after the spectral estimate had been obtained, without incorporating the expected LF/HF assignment into the optimization of any method.

The three spectral estimators exhibited similar overall agreement with the predefined stimulus-level spectral tendencies. MUSIC yielded the highest overall agreement (57.25%), followed by Welch (55.56%) and Lomb–Scargle (53.38%). The relatively small differences among the three methods indicate that the observed correspondence with the stimulus-level LF/HF hypothesis is not exclusive to the proposed MUSIC procedure.

The agreement was not uniform across stimuli. For the LF-expected stimuli, Welch and Lomb–Scargle frequently produced high agreement values. For example, Welch achieved 100% agreement for Disgust (Stimulus 6), 95.65% for Surprise (Stimulus 18), and 95.65% for Happiness (Stimulus 13). Similarly, Lomb–Scargle achieved 100% agreement for Disgust (Stimulus 6), 91.30% for Happiness (Stimulus 13), and 86.96% for several other LF-expected stimuli.

In contrast, the HF-expected stimuli generally exhibited lower agreement across all three estimators. For example, Amusement (Stimulus 12) yielded only 26.09%, 8.70%, and 4.35% agreement for MUSIC, Welch, and Lomb–Scargle, respectively. Similarly, Sadness (Stimulus 9) yielded 39.13%, 8.70%, and 13.04%, while Calmness (Stimulus 11) yielded 34.78%, 17.39%, and 21.74%, respectively. This consistent reduction in agreement for several HF-expected stimuli indicates that the observed discrepancy is not specific to MUSIC.

An additional example is Amusement (Stimulus 3), for which MUSIC obtained 73.91% agreement with the expected LF band, whereas Welch and Lomb–Scargle yielded 0%. This result illustrates that the dominant frequency estimated by the different spectral methods can differ substantially for individual stimuli, even when the same underlying HRV recordings are analyzed.

Overall, these results do not demonstrate that MUSIC is uniquely capable of recovering the predefined stimulus-level LF/HF tendency. Rather, the three methods produced broadly comparable aggregate agreement, with MUSIC showing a modestly higher overall value. The stimulus-level variability further indicates that the correspondence between the dominant spectral frequency and the a priori LF/HF hypothesis depends on both the stimulus and the spectral estimation method.

The values reported in this table represent a stimulus-level summary of independently processed participant recordings. The model order was selected separately for each recording before the dominant frequency was obtained. The subsequent averaging of $p^{*}$ and $f_{\mathrm{dom}}$ was performed only for descriptive stimulus-level analysis. Therefore, the reported agreement percentages should be interpreted as an independent post-selection evaluation of the a priori spectral hypothesis rather than as an optimization target or as evidence of a direct mapping between emotional state and autonomic physiological activity.

### 3.7. Controlled Synthetic Validation of Spectral Resolution

To independently assess the spectral behavior of the MUSIC estimator, controlled synthetic signals with known frequency components were analyzed. This validation was designed to separate the numerical spectral-resolution properties of the estimator from the physiological interpretation of the DREAMER recordings.

First, single-tone signals with known frequencies were evaluated for durations of 60, 120, and 180 s and signal-to-noise ratios (SNRs) of −5, 0, 10, and 20 dB. The frequency estimation error decreased as the SNR and signal duration increased. For the proposed signal-driven criterion, the mean absolute frequency error ranged from approximately 0.074–0.114 Hz at low SNR conditions to 0.0026–0.0058 Hz at 20 dB, depending on signal duration. The corresponding resolution success rate increased from approximately 12.5–16.3% at −5 dB to 85.0–96.3% at 20 dB. These results indicate that the accuracy of the MUSIC-based procedure is strongly dependent on signal quality and observation duration.

A more stringent controlled test was subsequently performed using a two-tone signal containing two known components at $f_{1}=0.10\;\mathrm{Hz}$, and $f_{2}=0.20\;\mathrm{Hz}$, with the two components separated by 0.10Hz. The test included 60, 120, and 180 s signal durations, SNRs of −5, 0, 10, and 20 dB, and 30 independent realizations for each condition, resulting in 360 two-tone test conditions.

For MUSIC, the known-order experiment evaluated every candidate model order p∈{2,…,10} independently for each synthetic condition. Consequently, 3240 MUSIC model order evaluations were performed. This analysis was deliberately separated from the adaptive model order selection problem: it evaluates the intrinsic ability of MUSIC to resolve the two known components for different values of *p*, whereas the adaptive methods subsequently determine *p* from the signal itself.

Resolution success was defined as the detection of two spectral peaks corresponding to the two known components within the predefined frequency-tolerance criterion. The resulting two-tone resolution statistics are summarized in Table 7.

Across all candidate MUSIC orders, the two-tone resolution success rate was 6.57%, with a mean of 1.088 detected components per test. The resolution therefore depended strongly on the selected model order, signal-to-noise ratio, and recording duration. In contrast, the adaptive signal-driven criterion selected an order that resolved both components in 0% of the 360 test conditions. This result indicates that the proposed model order selection criterion did not maximize two-tone spectral resolution under the controlled synthetic conditions. The other adaptive model order criteria also showed limited resolution, with AIC reaching 8.06%, whereas MDL, ESTER, eigengap, and the proposed criterion achieved 0% resolution.

The conventional spectral estimators provided substantially stronger two-tone resolution under the same controlled conditions. Welch resolved the two components in 92.78% of the 360 tests, while Lomb–Scargle achieved 100% resolution. Their mean numbers of detected components were 1.983 and 2.000, respectively.

The dependence of MUSIC resolution on *p* was particularly evident under favorable conditions. At 60 s and 20 dB, the resolution success rate increased substantially for larger model orders, reaching 73.3% for p=10. At 120 s and 20 dB, it reached 90.0% for p=10, while at 180 s and 20 dB it reached 93.3%. Thus, MUSIC can resolve the two known components under favorable conditions, but the result cannot be attributed to MUSIC independently of model order selection.

These controlled results demonstrate that MUSIC is capable of resolving closely spaced spectral components under favorable conditions, but that its resolution is highly sensitive to the selected model order, SNR, and signal duration. The experiment therefore does not support the use of a universal fixed model order as a guarantee of two-component spectral resolution. More importantly, it provides an independent validation of the distinction between the intrinsic resolution capability of MUSIC and the separate problem of automatically selecting an adequate model order.

Accordingly, the synthetic experiment is interpreted as a validation of the estimator’s numerical behavior rather than as evidence of physiological LF/HF discrimination. No DREAMER emotional labels, LF/HF assignments, valence, arousal, or dominance information were used in the synthetic model order evaluation.

### 3.8. Temporal Variation in the MUSIC Model Order

After the recording-level benchmark, a complementary window-based analysis was performed to examine whether the selected MUSIC model order remained stable throughout an individual HRV recording. This analysis was intended only to characterize the temporal behavior of the signal-driven model order criterion and was not used for physiological LF/HF classification.

For each HRV recording, the interpolated and filtered signal was divided into 30 s temporal windows with 50% overlap. The signal-driven model order criterion was independently evaluated within each window for all candidate orders p∈{2,…,10}. For each window *w*, the locally selected model order was defined as

(16) $$p^{*}\left(w\right)=\arg\min_{p\in \{2,\ldots ,10\}}J_{\mathrm{SD}}\left(p,w\right),$$

where $J_{\mathrm{SD}}\left(p,w\right)$ denotes the signal-driven criterion evaluated for candidate order *p* within temporal window *w*.

The resulting sequence $p^{*}\left(w\right)$ was used to identify temporal changes in the model order associated with variations in the signal-derived spectral structure. A representative model order for the complete recording was obtained as the most frequently occurring value among the window-level solutions. This procedure provides a recording-level summary while preserving information about local temporal variations in the selected model order.

**Figure 9:**
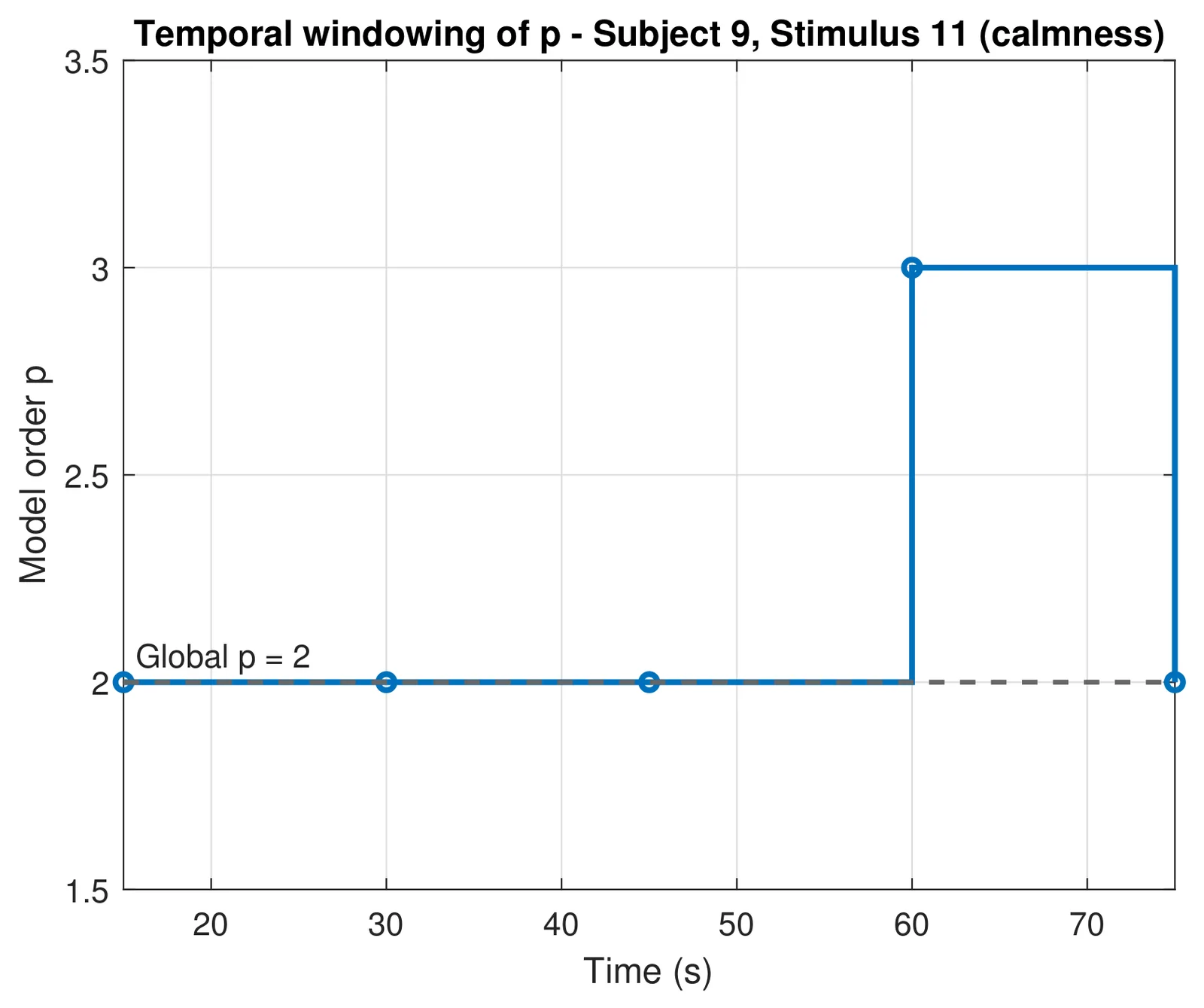
Temporal sequence of the signal-driven MUSIC model order $p^{*}\left(w\right)$ obtained for Subject 9 under Stimulus 11 (calmness). The HRV signal was analyzed using 30 s windows with 50% overlap. The dashed horizontal line indicates the most frequently selected window-level model order. The analysis is presented only as an exploratory assessment of temporal model order stability and does not involve LF/HF classification.

As a supplementary analysis, the signal-driven model order procedure was also applied to overlapping temporal windows of a representative HRV recording. This analysis was performed only to examine the temporal stability of the selected model order $p^{*}$. It was not used for the principal spectral analysis, model order selection of the 60 s recording, or LF/HF physiological interpretation. Therefore, the temporal sequence shown in Figure 9 should be interpreted exclusively as an exploratory assessment of model order stability.

Figure 9 illustrates this procedure for Subject 9 under Stimulus 11 (calmness). In this representative recording, the selected model order remained predominantly at p=2, with a local increase to p=3 in the final portion of the recording. Thus, the window-level analysis indicates local temporal variation in the selected model order within the recording.

This example illustrates that the signal-driven criterion can produce locally different model orders within the same HRV recording. Such temporal variation is consistent with the non-stationary nature of HRV and indicates that a single model order may not necessarily provide the same spectral configuration throughout an entire recording. Importantly, this window-based analysis was used only to evaluate temporal model order stability; no LF/HF assignment, emotional label, valence, arousal, or dominance information was used in the window-level optimization.

### 3.9. Distribution of the Optimized Model Order Across Emotional Stimuli

Following the signal-level optimization procedure, the representative model order associated with each emotional stimulus was identified by analyzing the distribution of optimized *p* values across all participant recordings. For each emotion, the optimized order selected for every signal was examined, and the most frequently occurring value was taken as the representative model order for that emotional condition. This representation provides a compact summary of the spectral configuration most consistently selected by the optimization framework and facilitates the comparison of dominant HRV spectral behavior across emotional stimuli. In addition, the mean dominant frequency obtained for each emotion is reported to support the physiological interpretation of the resulting LF- and HF-related patterns.

As shown in Table 8, the optimized model order exhibited different descriptive distributions across the analyzed emotional stimuli. Calmness, amusement, happiness, and sadness were more frequently associated with lower model orders, whereas surprise, fear, excitement, disgust, and anger more frequently showed higher selected orders. These observations are descriptive and should not be interpreted as evidence of a deterministic relationship between emotional category and MUSIC model order.

### 3.10. Distribution of Model Order Across Emotional Conditions

To further characterize the behavior of the optimized model order within each emotional condition, descriptive statistics of the selected *p* values were computed across all participant recordings. For every emotion, the distribution of optimized model orders was summarized in terms of mean, median, standard deviation, minimum, and maximum values. This analysis provides a descriptive view of the variability of the selected model order across emotional conditions, including differences in the dispersion of the selected values across participant recordings.

The descriptive statistics in Table 9 show differences in the distribution of the selected model order across emotional stimuli. Calmness, amusement, happiness, and sadness showed mean and median values close to p=2, whereas surprise, fear, excitement, disgust, and anger showed values centered closer to p=4. The observed dispersion also differed across conditions. These results are descriptive and do not establish a statistically significant effect of emotional condition on the selected model order.

## 4. Discussion

The results obtained in this study show that HRV spectral characterization using MUSIC depends strongly on model order selection, and that this choice has direct implications for the physiological interpretation of autonomic modulation under emotional stimulation. The preliminary empirical evaluation showed that different values of *p* may shift the dominant frequency between different spectral regions, potentially modifying the subsequent interpretation of the HRV response. In this sense, the empirical stage should not be interpreted as a final selection criterion, but rather as evidence that the spectral response of MUSIC exhibits a nontrivial dependence on model order and therefore requires an explicit optimization strategy.

The proposed signal-driven model order selection framework addressed this issue by integrating information-theoretic criteria, signal-subspace characteristics, eigengap information, and model-complexity penalization into a unified selection criterion. Importantly, LF/HF physiological information and emotional labels were not used during model order selection and were considered only for post-selection evaluation. The results showed that the proposed criterion produced a recording-dependent distribution of model orders, with p=4 being the most frequently selected model order, rather than imposing a single fixed order across the entire dataset. This behavior supports the use of a signal-driven model order selection strategy for MUSIC-based HRV spectral characterization.

At the stimulus level, differences were observed in the distribution of the selected model orders. These differences should be interpreted descriptively and do not establish a deterministic relationship between emotional category and MUSIC model order.

Another relevant finding was the temporal variability of the optimized order within the same recording. The window-based analysis showed that *p* did not necessarily remain constant throughout the signal, but could vary across consecutive temporal segments, even under the same emotional stimulus. This behavior suggests that HRV under emotional stimulation should not be assumed to be strictly stationary, and that the spectral structure captured by MUSIC may evolve over time. Within this context, the CWT served as a complementary time–frequency reference for the illustrative calmness case, providing a complementary reference for the localization of the dominant frequency within the HF band. However, the CWT was not used for order selection or global evaluation but rather as a visual support for interpreting the temporal distribution of spectral energy.

Several limitations should also be considered. The analysis was performed on a controlled database with 23 participants and 18 predefined audiovisual stimuli, so the observed variability remains conditioned by the experimental characteristics of DREAMER. A further methodological limitation concerns R-peak detection. The minimum inter-peak distance of 0.60 s corresponds to approximately 100 bpm, which may result in missed consecutive R-peaks at higher heart rates. This effect may vary across participants because of inter-individual differences in cardiac responses. Although RR intervals underwent the described quality-control and correction procedure, the R-peak detector was not independently validated on the DREAMER recordings. Potentially undetected R-peaks should therefore be considered when interpreting the subsequent HRV spectral results.

In addition, the expected band assigned to each stimulus was used only as a post-selection physiological reference and should be interpreted as a theoretical tendency rather than as an absolute ground truth for every subject.

### Implications of Model Order Selection for MUSIC Resolution

The results demonstrate that the model order parameter *p* is a critical component of MUSIC-based HRV spectral analysis. The analysis of the complete DREAMER dataset showed that the selected model order depends on the criterion used for model order determination. In particular, AIC and MDL systematically selected the upper limit of the evaluated range (p=10), whereas ESTER and eigengap favored lower model orders. The proposed signal-driven criterion produced a recording-dependent distribution concentrated around p=4. Therefore, the use of a fixed model order should be regarded as an empirical choice rather than as an intrinsic property of the MUSIC estimator.

The controlled synthetic validation further clarifies this observation. The two-tone experiment demonstrated that the ability of MUSIC to resolve two closely spaced components depends strongly on the selected model order, as well as on signal duration and SNR. Although higher model orders improved the probability of resolving the two components under favorable conditions, this behavior was not reproduced consistently at lower SNRs or shorter observation durations. Consequently, increasing *p* cannot be considered a general solution for improving spectral resolution.

An important finding is that the proposed signal-driven model order criterion did not maximize two-tone resolution in the controlled experiment. This result does not invalidate its use for the DREAMER recordings; rather, it clarifies the objective of the proposed strategy. The criterion was designed to select a stable model order from characteristics derived from the analyzed HRV signal, without using physiological labels, expected LF/HF assignments, or emotional information. Thus, its objective is recording-dependent model selection rather than optimization of a known two-component spectral separation problem.

The synthetic experiment also establishes an important limitation of the present approach. A model order that is appropriate for the spectral characteristics of an individual HRV recording cannot be assumed to provide maximum resolution for every possible pair of closely spaced sinusoidal components. In particular, the zero two-tone resolution obtained with the proposed adaptive criterion indicates that additional information or a different objective function would be required if explicit resolution of multiple known components were the primary goal.

These findings support the use of the proposed strategy as a signal-driven model order selection procedure while avoiding the stronger claim that it provides universally optimal MUSIC resolution. The predominance of p=4, together with the selection of other model orders in a smaller proportion of recordings, further indicates that the selected order remains recording-dependent. The results therefore motivate future extensions in which resolution-oriented terms could be incorporated into the optimization objective and evaluated independently from physiological interpretation. Such extensions would require additional validation to determine whether improved numerical resolution translates into more reliable characterization of the spectral components present in short-duration HRV recordings.

A further limitation concerns the magnitude and interpretation of the observed LF/HF agreement. The overall agreement obtained after signal-driven model order selection was 57.25%, which is relatively close to the 50% level expected for a binary classification problem. Because the expected LF/HF assignments are theoretical stimulus-level hypotheses rather than independently measured physiological ground truth, this agreement should not be interpreted as classification accuracy or as evidence of methodological superiority. In addition, no fixed-order baseline was used to establish a statistical improvement in spectral stability. Accordingly, the present results should be regarded as descriptive evidence of model order sensitivity rather than definitive evidence of improved spectral estimation performance.

## 5. Conclusions

This work presented an HRV spectral characterization framework based on the MUSIC algorithm and a signal-driven model-order selection strategy applied to ECG signals from the DREAMER database. The proposed methodology integrates information-theoretic criteria, signal-subspace characteristics, eigengap information, and model-complexity penalization without incorporating LF/HF physiological information or emotional labels into the model-order selection process.

The results showed that model order selection influences the resulting dominant frequency localization and therefore can affect the spectral characterization of HRV. Across the complete DREAMER dataset, the signal-driven procedure produced a recording-dependent distribution of selected model orders, with p=4 being the most frequently selected order and p=3 occurring in a smaller proportion of recordings. The subsequent stimulus-level LF/HF evaluation showed non-uniform agreement across emotional stimuli, indicating that the predefined spectral tendency cannot be regarded as a deterministic physiological outcome.

Taken together, these findings support the use of signal-driven model order selection as a framework for examining the sensitivity of MUSIC-based HRV spectral characterization to the model order parameter. However, the present results do not establish a statistically significant improvement over fixed-order or conventional spectral estimators, nor do they demonstrate that the predefined LF/HF tendencies constitute physiological ground truth. Further validation using independent physiological measurements, larger datasets, and participant-aware statistical analyses is required to determine whether the proposed strategy provides a measurable performance advantage.

## Figures and Tables

**Table 1:** DREAMER stimuli, VAD ratings, valence–arousal classification, and expected HRV spectral bands.

ID	Film Clip	Emotion	Val.	Aro.	Dom.	Quadrant	Expected Band
1	Searching for Bobby Fischer	Calmness	3.17	2.26	2.09	HV–LA	HF
2	D.O.A.	Surprise	3.04	3.00	2.70	HV–NA	HF
3	The Hangover	Amusement	4.57	3.83	3.83	HV–HA	LF
4	The Ring	Fear	2.04	4.26	4.13	LV–HA	LF
5	300	Excitement	3.22	3.70	3.52	HV–HA	LF
6	Van Wilder	Disgust	2.70	3.83	4.04	LV–HA	LF
7	Wall-E	Happiness	4.52	3.17	3.57	HV–HA	LF
8	Crash	Anger	1.35	3.96	4.35	LV–HA	LF
9	My Girl	Sadness	1.39	3.00	3.48	LV–NA	HF
10	The Fly	Disgust	2.17	3.30	3.61	LV–HA	LF
11	Pride and Prejudice	Calmness	3.96	1.96	2.61	HV–LA	HF
12	Modern Times	Amusement	3.96	2.61	2.70	HV–LA	HF
13	Remember the Titans	Happiness	4.39	3.70	3.74	HV–HA	LF
14	Gentleman’s Agreement	Anger	2.35	2.22	2.39	LV–LA	HF
15	Psycho	Fear	2.48	3.09	3.22	LV–HA	LF
16	The Bourne Identity	Excitement	3.65	3.35	3.26	HV–HA	LF
17	Shawshank Redemption	Sadness	1.52	3.00	3.96	LV–NA	HF
18	The Departed	Surprise	2.65	3.91	3.57	LV–HA	LF

**Table 2:** Theoretical mapping between emotional states, dimensional affect, and autonomic nervous system (ANS) patterns based on the established psychophysiological literature. The associations represent general trends.

Emotion	Valence	Arousal	Dominance	Predominant ANS Pattern	References
Calmness	High	Low	High	Parasympathetic dominance	McCraty (2014) [22]
Sadness	Low	Low	Low	Parasympathetic withdrawal/reduced activation	Kreibig (2010), Gross (1998) [23,24]
Happiness	High	Moderate	High	Balanced ANS with parasympathetic engagement	McCraty (2014), Bradley & Lang (1994) [22,25]
Amusement	High	Moderate–High	Medium	Dynamic autonomic activation	Kreibig (2010), Shiota et al. (2014) [23,26]
Fear	Low	High	Low	Increased autonomic activation	Kreibig (2010), Lang et al. (1993) [23,27]
Anger	Low	High	High	Increased autonomic activation and cardiovascular reactivity	Kreibig (2010), Stemmler (2004) [23,28]
Disgust	Low	Medium–High	Medium	Increased autonomic reactivity with complex autonomic modulation	Kreibig (2010), Rozin & Fallon (1987) [23,29]
Surprise	Neutral	High (transient)	Medium	Autonomic co-activation (sympathetic + parasympathetic)	Kreibig (2010), Reisenzein (2000) [23,30]
Excitement	High	Very High	High	Sympathetic activation with strong variability	Bradley and Lang (1994); Lang et al. (1993) [25,27]

**Table 3:** Comparison of MUSIC model order selection strategies across the complete DREAMER dataset (N=414 participant–stimulus recordings).

Method	Mean *p*	SD *p*	Median *p*	Min.	Max.	Mean $f_{\mathrm{dom}}$ (Hz)
Fixed p=4	4.000	0.000	4	4	4	0.1168
AIC	10.000	0.000	10	10	10	0.1048
MDL	10.000	0.000	10	10	10	0.1048
ESTER	2.942	0.818	3	2	4	0.1625
Eigengap	3.367	0.686	3	2	4	0.1613
Proposed	3.659	0.490	4	3	5	0.1514

**Table 4:** Distribution of the model orders selected by the proposed signal-driven criterion across the DREAMER recordings.

Model Order	Number of Recordings	Percentage (%)
p=3	144	34.78
p=4	267	64.49
p=5	3	0.72
Total	414	100.00

**Table 5:** Stimulus-level evaluation of the proposed signal-driven MUSIC model order selection against the expected HRV spectral band. The expected LF/HF band was defined a priori from the DREAMER stimulus hypothesis. The model order was selected independently of this information, and band agreement was evaluated only after obtaining the dominant frequency.

ID	Emotion	Expected Band	Mean $p^{*}$	Mean $f_{\mathrm{dom}}$ (Hz)	LF (%)	HF (%)	Agreement (%)
1	Calmness	HF	3.522	0.1647	52.17	47.83	47.83
2	Surprise	HF	3.565	0.1632	52.17	47.83	47.83
3	Amusement	LF	3.739	0.1287	73.91	26.09	73.91
4	Fear	LF	3.609	0.1506	60.87	39.13	60.87
5	Excitement	LF	3.739	0.1359	69.57	26.09	69.57
6	Disgust	LF	3.870	0.1199	82.61	17.39	82.61
7	Happiness	LF	3.696	0.1423	69.57	30.43	69.57
8	Anger	LF	3.826	0.1406	73.91	26.09	73.91
9	Sadness	HF	3.565	0.1520	56.52	39.13	39.13
10	Disgust	LF	3.696	0.1646	65.22	34.78	65.22
11	Calmness	HF	3.696	0.1619	65.22	34.78	34.78
12	Amusement	HF	3.739	0.1447	73.91	26.09	26.09
13	Happiness	LF	3.739	0.1410	73.91	26.09	73.91
14	Anger	HF	3.522	0.1585	52.17	43.48	43.48
15	Fear	LF	3.609	0.1596	56.52	43.48	56.52
16	Excitement	LF	3.739	0.1398	73.91	26.09	73.91
17	Sadness	HF	3.522	0.1624	52.17	47.83	47.83
18	Surprise	LF	3.478	0.1948	43.48	56.52	43.48
**Overall **			3.659	0.1514	63.77	35.51	57.25

**Table 6:** Stimulus-level comparison of LF/HF band agreement obtained with the proposed signal-driven MUSIC procedure, Welch’s method, and the Lomb–Scargle periodogram. The expected LF/HF band was defined a priori from the DREAMER stimulus hypothesis. For MUSIC, the model order was selected independently of the expected band. For all three methods, LF/HF agreement was evaluated only after obtaining the dominant frequency.

ID	Emotion	Expected Band	$p^{*}$	MUSIC Agreement (%)	Welch Agreement (%)	Lomb–Scargle Agreement (%)
1	Calmness	HF	3.522	47.83	17.39	17.39
2	Surprise	HF	3.565	47.83	13.04	17.39
3	Amusement	LF	3.739	73.91	0.00	0.00
4	Fear	LF	3.609	60.87	91.30	82.61
5	Excitement	LF	3.739	69.57	86.96	86.96
6	Disgust	LF	3.870	82.61	100.00	100.00
7	Happiness	LF	3.696	69.57	91.30	86.96
8	Anger	LF	3.826	73.91	91.30	86.96
9	Sadness	HF	3.565	39.13	8.70	13.04
10	Disgust	LF	3.696	65.22	78.26	69.57
11	Calmness	HF	3.696	34.78	17.39	21.74
12	Amusement	HF	3.739	26.09	8.70	4.35
13	Happiness	LF	3.739	73.91	95.65	91.30
14	Anger	HF	3.522	43.48	17.39	30.43
15	Fear	LF	3.609	56.52	86.96	73.91
16	Excitement	LF	3.739	73.91	86.96	82.61
17	Sadness	HF	3.522	47.83	13.04	13.04
18	Surprise	LF	3.478	43.48	95.65	82.61
**Overall **			3.659	57.25	55.56	53.38

**Table 7:** Controlled two-tone spectral-resolution validation for $f_{1}=0.10$ Hz and $f_{2}=0.20$ Hz. The experiment comprised 360 independent two-tone conditions. MUSIC was evaluated at all nine candidate model orders, resulting in 3240 MUSIC evaluations.

Method	Evaluations	Resolution (%)	Mean $N_{\mathrm{detected}}$	RMSE (Hz)
MUSIC, p=2:10	3240	6.574	1.088	0.00631
AIC	360	8.056	1.089	0.00780
MDL	360	0.000	1.000	–
ESTER	360	0.000	1.000	–
Eigengap	360	0.000	1.000	–
Proposed	360	0.000	1.000	–
Welch	360	92.778	1.983	0.00276
Lomb–Scargle	360	100.000	2.000	0.00033
AR–Burg	360	0.000	1.000	–

**Table 8:** Representative optimized model order and mean dominant frequency obtained for each emotional stimulus. The representative order corresponds to the most frequently selected optimized *p* value across all participant recordings for the corresponding emotion.

Emotion	Representative *p*	Mean Dominant Frequency (Hz)	Expected Band
Calmness	2	0.1887	HF
Surprise	4	0.1328	LF
Amusement	2	0.1841	HF
Fear	4	0.1379	LF
Excitement	4	0.1332	LF
Disgust	4	0.1296	LF
Happiness	2	0.1867	HF
Anger	4	0.1369	LF
Sadness	2	0.1873	HF

**Table 9:** Descriptive statistics of the optimized model order *p* obtained for each emotional stimulus across all participant recordings.

Emotion	Mean	Median	SD	Min	Max	Mode
Calmness	2.02	2	0.15	2	3	2
Surprise	4.30	4	0.84	2	6	4
Amusement	2.00	2	0.00	2	2	2
Fear	4.13	4	0.50	4	6	4
Excitement	4.39	4	0.80	4	6	4
Disgust	4.39	4	0.80	4	6	4
Happiness	2.02	2	0.15	2	3	2
Anger	4.17	4	0.71	2	6	4
Sadness	2.02	2	0.15	2	3	2

## Data Availability

The data analyzed in this study are publicly available from the DREAMER database described by Katsigiannis and Ramzan in “DREAMER: A Database for Emotion Recognition Through EEG and ECG Signals from Wireless Low-cost Off-the-Shelf Devices” (IEEE Journal of Biomedical and Health Informatics, 2018).
